# Synthetic Microbial Ecology: Engineering Habitats for Modular Consortia

**DOI:** 10.3389/fmicb.2017.01125

**Published:** 2017-06-16

**Authors:** Sami Ben Said, Dani Or

**Affiliations:** Department of Environmental Systems Science, Soil and Terrestrial Environmental Physics, ETH ZürichZürich, Switzerland

**Keywords:** synthetic ecology, microbial ecology, microbial consortia, consortia assembly, modular consortia, engineering consortia, engineering habitats

## Abstract

The metabolic diversity present in microbial communities enables cooperation toward accomplishing more complex tasks than possible by a single organism. Members of a consortium communicate by exchanging metabolites or signals that allow them to coordinate their activity through division of labor. In contrast with monocultures, evidence suggests that microbial consortia self-organize to form spatial patterns, such as observed in biofilms or in soil aggregates, that enable them to respond to gradient, to improve resource interception and to exchange metabolites more effectively. Current biotechnological applications of microorganisms remain rudimentary, often relying on genetically engineered monocultures (e.g., pharmaceuticals) or mixed-cultures of partially known composition (e.g., wastewater treatment), yet the vast potential of “microbial ecological power” observed in most natural environments, remains largely underused. In line with the Unified Microbiome Initiative (UMI) which aims to “discover and advance tools to understand and harness the capabilities of Earth's microbial ecosystems,” we propose in this concept paper to capitalize on ecological insights into the spatial and modular design of interlinked microbial consortia that would overcome limitations of natural systems and attempt to optimize the functionality of the members and the performance of the engineered consortium. The topology of the spatial connections linking the various members and the regulated fluxes of media between those modules, while representing a major engineering challenge, would allow the microbial species to interact. The modularity of such spatially linked microbial consortia (SLMC) could facilitate the design of scalable bioprocesses that can be incorporated as parts of a larger biochemical network. By reducing the need for a compatible growth environment for all species simultaneously, SLMC will dramatically expand the range of possible combinations of microorganisms and their potential applications. We briefly review existing tools to engineer such assemblies and optimize potential benefits resulting from the collective activity of their members. Prospective microbial consortia and proposed spatial configurations will be illustrated and preliminary calculations highlighting the advantages of SLMC over co-cultures will be presented, followed by a discussion of challenges and opportunities for moving forward with some designs.

## 1. Microbial consortia: efficient and metabolically versatile associations

Microbes are present in all environments on our planet and are often forced to interact within close proximity and share resources. Consequently, microbial life in natural systems occurs in a concourse, where the interactions between its members are key to their survival (Brenner et al., 2008; Stewart, 2012; Jagmann and Philipp, 2014). Even the simplest characterized consortia may contain from ten to thousands of species (Curtis et al., 2002).

A consortium, due to the multiple species involved, possesses a larger pool of genes than monocultures. That resulting diversity in metabolic pathways allows a consortium to perform more complex tasks than single organisms, while utilizing the resources available in its environment more efficiently (Sun and Cheng, 2002; Fu et al., 2009). Consortia can therefore use simpler, less refined substrates (e.g., whey, molasses,..) and display a higher bioconversion efficiency (higher yields) than monocultures, while requiring a less expensive purification process (Sabra et al., 2010; Zhang and Wang, 2016). Moreover, its members interact by exchanging signals or trading metabolites and this enable the coordination of their activity, which is especially relevant for multistep-processes like degradation of complex biological material (Brenner et al., 2008; Bader et al., 2010; Bernstein and Carlson, 2012; Hays et al., 2015). The result of this division of labor, besides reducing potential interferences between bioprocesses and offering a cellular environment adapted to the requirements of each biochemical process, is a reduced biosynthetic load and metabolic stress for every microbial strain (Zhang and Wang, 2016). Through the formation of biofilms, microbial consortia show an increased robustness to environmental perturbations and are more resistant to invasion by other microorganisms, than single species (Burmolle et al., 2006; Hays et al., 2015). Therefore, microbial consortia could provide an answer to some of the drawbacks that the pure cultures of single, often genetically engineered, microbial strains employed in many biotechnological processes suffer from Jagmann and Philipp (2014).

Microbial consortia have been employed as mixed-cultures for thousands of years for the preparation of traditional beverages and food production by fermentation, and more recently for wastewater treatment, composting, bioremediation, biomining and biofuel production (Daims et al., 2006; Kleerebezem and van Loosdrecht, 2007; Angenent and Wrenn, 2008; Sabra et al., 2010; Brune and Bayer, 2012; Zuroff and Curtis, 2012; He et al., 2013). As artificial consortia are composed of microorganisms that have been selected to perform a particular task, their applications are therefore more specific and often ranges from the production of enzymes, fine chemicals, biopolymers, food additives, antimicrobials, to bioelectricity with microbial fuel cells (Lynd et al., 2002; Bader et al., 2010; Sabra et al., 2010; Bernstein and Carlson, 2012; Zuroff and Curtis, 2012). While a few studies reported constructing consortia of more than two microbial species (Kim et al., 2008), the vast majority of current applications employ binary cultures. These co-cultures, where the different species are exposed to the same conditions (nutrients, T°, pH, [O_2_],..) as they grow in a common medium, are limited by the need for environmental conditions compatible for each member. Besides some isolated examples such as bioremediation (Bernstein and Carlson, 2012), biofuels (ethanol, isobutanol) and chemicals (lactic acid, acetate), synthetic microbial consortia are rarely used in biotechnology and mostly belong to the realm of academic research. Although genetically engineered consortia hold promise for future industrial applications, they will require further development to serve as bioprocessing platforms (Bernstein and Carlson, 2012).

Industrial applications of microbial consortia remain rudimentary as they mostly rely on co-cultures or mixed-cultures, and as stated by the Unified Microbiome Initiative (Alivisatos et al., 2015), much improvement will be required to fully exploit the potential offered by microorganisms (Konopka, 2009; Sabra et al., 2010; Konopka et al., 2015; Lindemann et al., 2016). We hypothesize that a higher level of environmental design will provide the spatial control necessary to construct microbial consortia in a more predictable and flexible manner. Here, we propose to construct spatially linked microbial consortia (SLMC) and define their spatial organization to provide each microorganism with optimal environmental conditions, while connecting them to enable interactions, in order to fulfil a desired biochemical function. In one scenario the system will rely on diffusion mediated communication, in another, exchanges will occur by controlled (convective) fluxes of media and intermediate products between the different members of the consortium. By engineering interconnected modules fulfilling a specific biochemical function, we will reduce the need for compatible growth conditions, thereby considerably increasing the potential combinations of microorganisms and hence their applications. In nature, one obvious example of this compartmentalization strategy, is the presence of many types of organelles found in eukaryotic cells (ER, golgi apparatus, mitochondria, vacuole, nucleus, chloroplasts, lysosome, peroxisome,..), whose roles are to provide appropriate conditions and confine the specific function they accomplish (Alberts et al., 2008).

## 2. Engineering spatially linked microbial consortia (SLMC)

The concept of engineering the environment in which interacting microbial species grow is not new. In 1975, Tannenbaum et al. designed a “multiple diffusion chamber” (US Patent) which allows several microorganisms to grow in separate chambers while sharing a common medium through which they can communicate by exchanging soluble molecules by diffusion (Tannenbaum and Kornfeld, 1975). In 1999, a membrane-partitioned glass vessel for dialysis culture was designed by Ohno et al. to study the interactions between two symbiotic bacteria (Ohno et al., 1999). Similar works from Ueda et al. (2002) as well as Dietz et al. (2013) have both used the dialysis membrane reactor from Pörtner and Märkl (1998), to culture two microbial species on each side of a membrane that allows the exchange of low-molecular-mass components. A limitation of these platforms is that they offer a single set of environmental conditions to the cultured microbes. Although the microorganisms were spatially isolated their communication relied on the diffusion of molecules they secrete into the shared medium. In order to find a solution for easier and more accurate quantitative study of the kinetics of microbial growth in co-cultures, Salgado et al. built in 1998, a two-reservoir, hollow-fiber bioreactor (Salgado et al., 1998). They showed that it is possible to obtain the same growth conditions as in co-culture by growing both species in separate bioreactors and controlling the fluxes of the medium exchanged between the bioreactors in a bidirectional manner. This allowed them to apply to each microbial species, analytical methods designed for monocultures that can so far not be applied to co-cultures (e.g., OD,..). The precise measure of quantitative interaction dynamics between microbial species is only possible by the measure of the growth kinetics of each species in pure culture, as well as in co-culture (Frederickson, 1977), and this knowledge is of high importance for the control of cultures using microbial consortia as required in the industry. The milestone that this study represents is not only due to the ability to separate growing interacting microbial species, but comes from its intrinsic property which allows not only to control the interspecies communication (fluxes of medium) but also the environment in which each species is cultured. Therefore, in spite of the many advantages that synthetic biology offers, our work will mostly aim at engineering the environment to define the conditions in which the different species will grow and control their interactions. Each microorganism could thus be provided with a more suitable and controllable environment, conceivably resulting in enhanced activity and productivity.

### 2.1. SLMC: spatially linked microbial consortia

**Figure 1:**
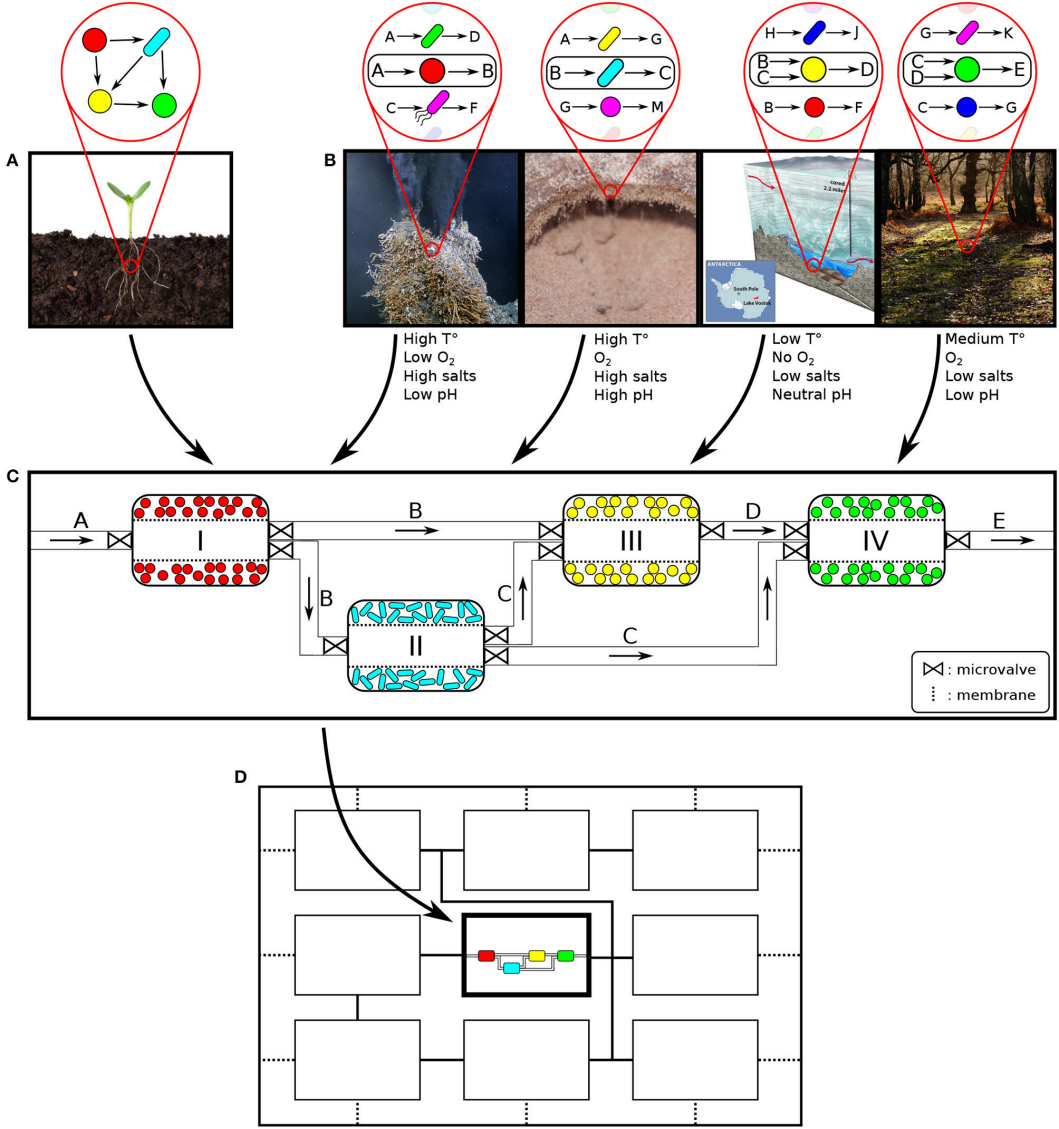
Conceptual overview and design of a spatially linked microbial consortium (SLMC). **(A)** Natural microbial consortium. **(B)** Artificial microbial consortium: selection of the members based on their ability to accomplish part of a bioprocess of interest (convert substrate A to product E). The reduced need for compatible environmental conditions that SLMC offers, would allow the combination of microbial species with incompatible requirements. This would enable the construction of *de novo* consortia (not found in nature) resulting in new products or applications. Left to right: hydrothermal vent, desert biocrust, (sub-glacial) lake Vostok, and deciduous forest. **(C)** Each module offers different environments to promote a specific biochemical function. Connections between modules enable interactions. **(D)** The modularity of SLMC would allow to incorporate this microbial consortium (sub-consortium) as part of a larger biochemical network (super-consortia). Images sources: Flickr (seedling, hydrothermal vent: Ocean Networks Canada, lake Vostok: US National Science Foundation, forest: G. Crutchley) and Arizona State University, Estelle Couradeau (biocrust).

The main proposal of this study is to suggest a new approach to assemble microbial consortia based on optimizing local conditions for its members while linking metabolite exchanges in an “optimal” fashion. The hypothesis is that such constructed, or synthetic ecological system would overcome diffusional and connectivity limitations present in natural systems, optimize and enforce spatial self-organization, and introduce actors in desired proportions to shorten the time for attaining optimality and increase robustness. A potential strategy is to engineer spatially partitioned niches by segregating, in separate modules, the bioprocesses that require distinct environmental conditions, while connecting them to allow the microbial species to interact (Figure 1C).

This compartmentalization strategy would increase control over the system (consortium) such that each element would offer specific settings compatible with the consortium member. At the limit, each connected niche would host a single species which would allow us to tailor the growth conditions and thus regulate the growth of each member to optimize the functionality of the consortium. Such a degree of freedom would even allow the assembly of microbial species with incompatible requirements (aerobe/anaerobe, acidophile/alkalophile, thermophile/mesophile, halophile/non-halophile,..).

Moreover, by separating and concatenating the members of a microbial consortium, we reduce the average exposure of its members to the intermediate compounds and by-products secreted by the other species, which could potentially inhibit their growth or activity. In nature, where members of a microbial consortium grow together in close proximity, each microorganism can potentially impact the whole community. The spatial segregation suggested by SLMC would reduce that effect by making upstream elements independent of downstream ones, therefore preventing the exposure of upstream members to by-products of downstream members, and their potential inhibiting effects. This would result in an increased stability and predictability of the bioprocess, by reducing the effect of perturbations. This increased segregation has also a limiting effect on interspecies competition. In addition to the medium flowing from one module to the next, each functional element could be supplied with supplementary nutrients if those were depleted by upstream microorganisms. This “supply along the way” would basically avoid the competition for common substrates as observed in nature, and allow each strain to reach the population required to accomplish its step in the overall bioprocess. In a syntrophic relationship where inhibiting by-products of one species feed another, separation of such a mutualistic interaction could potentially be detrimental to both members. Should the tuning of the operating conditions not provide enough control to prevent by-products from reaching inhibiting concentrations, both microbial partners could be kept in the same module and grown as co-culture. A spatial element of control could nevertheless be included by segregating them with membranes allowing the exchange of metabolites by diffusion.

Ultimately, the hypothesis that optimization of specific biological functions, could be achieved via spatial control over fluxes and topology (connections) of microbial consortia members would be tested in experiments and compare performance to current biotechnological applications such as co-cultures. The higher level of spatial control, tailoring each microbial population and thus each biochemical processes could be tuned to achieve and maximize the desired overall function. Specifically, the objective would be to test a selected microbial consortium in two configurations, one that allows no spatial control, i.e., culturing them in co-culture where all the members will grow in a shared environment, and the other where the microbial species will be cultured in separate but interconnected environments, allowing the highest possible degree on spatial control to attempt to maximize the function of each module. The performance of the microbial consortium in both scenarios will be quantified and compared in terms of yield, whether it is in moles of a compound X in the effluent per moles of that same compound X in the influent (for a pollutant degrading consortium), or in grams/moles of product per grams/moles of substrate (for consortia converting a substrate that can easily be quantified), or in grams/moles of product per grams of biomass (for microbial consortia converting substrates that are more difficult to quantify, e.g., photoautotrophs consuming CO_2_ and light). Here, we will present some of the existing engineering tools available to control and organize the environment to culture microbial consortia as well as their populations and their interactions.

### 2.2. Selection of compatible consortium members

The members of an artificial microbial consortium would be selected for their ability to perform one or multiple steps of a desired biochemical process, and interact by passing to each other the intermediate products (Figure 1B). One could directly select from a natural microbial community the members that perform a desired bioprocess (Figure 1A). Their interactions would determine the level of segregation and the conditions of each module would be tuned to optimize their functionality (Figure 1C). Alternatively, when constructing an artificial microbial consortia (not found in nature) to perform a biochemical process, choosing partners that are bound to cooperate seems to be an obvious solution. Cooperative interactions, such as mutualism or commensalism, are commonly found in nature, even across kingdoms (e.g., plant-pollinator interaction, lichens,..) (Bronstein, 1994). At the microbial level, commensal relations, when one organism feeds on the metabolic waste of another are often observed (food chain, Bernstein and Carlson, 2012; Großkopf and Soyer, 2014, mutualism, Sabra et al., 2010). Trophic interactions such as mutualism or commensalism could therefore be good strategies to assemble an artificial microbial consortium. Compatibility does not imply similar environmental conditions, but rather that the selected members would each need to fulfill their part of the overall biochemical transformation.

### 2.3. Spatial layout of interactions and connections

**Figure 2:**
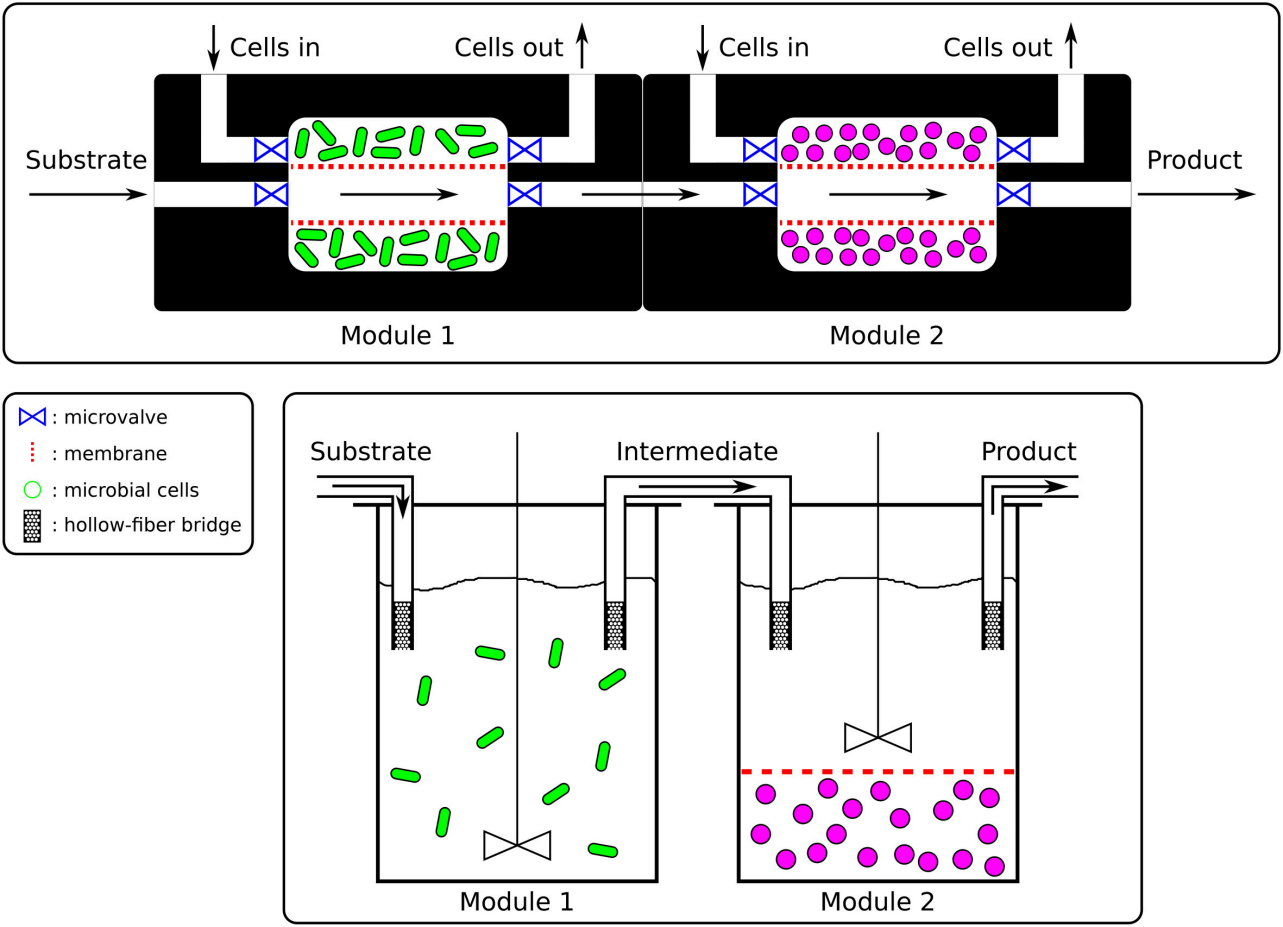
Microfluidics vs. Bioreactors. **(Top)** The microfluidic platform (nL to μL) would be made of PDMS and connecting channels would allow the exchange of media and intermediate products between the chambers as well as the replacement of the cells (here only represented on one side). The fluxes would be controlled by pumps and valves. Microbial species cultured in those chambers would be contained by membranes. **(Bottom)** In the sequential bioreactors platform (100 mL to hL), well-stirred bioreactors would be connected by hollow-fiber bridges and overhead pressure or pumps would allow the flow of media and intermediates between bioreactors. Cells could additionally be confined by membranes (right bioreactor) should this be necessary.

Once members of a consortium have been selected, their interactions will determine optimal spatial disposition. Members responsible for early steps of a biotransformation would be positioned upstream and the by-products of their metabolism (intermediate products) would be passed on to downstream members for further bioconversion. The SLMC concept would not be limited to any scale and could range from micrometers culture chambers to industrial size bioreactors. The scaling of the bioprocesses could be mainly accomplished in two ways, by parallelization (addition of multiple units in parallel) or by scaling-up (increasing the volume of the modules). Ultimately, the concept of combining the activities of multiple microbial species would be applicable at the micro- as well as at the macroscopic scale, assuming no scale-up issues linked to transport phenomena (mixing, oxygen transfer, heat transfer, dispersal of nutrients, acid, base,..), asepsis, genetic stability or downstream processing (separation, purification,..) (Charles, 1985; Reisman, 1993; Palomares and Ramirez, 2000; Villadsen et al., 2011). The requirements of a specific application rather than SLMC will determine the scale of the platform used. The spatial layout of the culture chambers or bioreactors and their connectivity will then need to be defined, based on the interaction motif of the microbial consortium at play. Whether the cells are cultured in bioreactors or in a microfluidic platform, connections between the modules would allow the microbial species to interact by controlled exchanges of media and intermediate products (Figure 2). Hollow fiber bridges would for example connect the bioreactors while keeping the cells confined to their respective batch, and avoid cross-contamination (Manjarrez et al., 2000). For microfluidics, ultra- or microfiltration membranes would keep the cells trapped in each chamber while allowing media and intermediates to be transferred downstream for their further processing (Figure 2) (Eykamp, 1995; Matson, 1995; van Reis and Zydney, 2001; Charcosset, 2006; van Reis and Zydney, 2007).

#### 2.3.1. Microfluidics

For the microfluidic platform one could envision using the popular microfabrication technique of soft lithography and the polymer PDMS (Kim et al., 2008; Frimat et al., 2011; Hong S. et al., 2012; Hong S. H. et al., 2012; Leung et al., 2012; Jeong et al., 2015; Luo et al., 2015; Mohan et al., 2015). Besides being a quick and inexpensive method, soft lithography would allow to easily construct the reservoirs in which the cells would grow, the potentially complex network of channels connecting those compartments and pattern their surface should this be needed (Duffy et al., 1998; Anderson et al., 2000; McDonald et al., 2000; Whitesides et al., 2001; McDonald and Whitesides, 2002). With the countless number of potential combinations of microorganisms that SLMC offers, and the multitude of layouts those would require, soft lithography and PDMS clearly present the required flexibility. Furthermore, the broad and tunable spectrum of physical properties offered by PDMS (Kuo, 1999; Lamberti et al., 2014) makes it a versatile polymer to accommodate the diversity of biological conditions needed to culture a variety of microorganisms (pH, temperature,..) (Becker and Gärtner, 2008; Ren et al., 2013). Moreover, the ease with which PDMS can be reversibly sealed (by simple contact), to another piece of PDMS, glass, or other substrates, befits the modularity of SLMC by facilitating the addition or replacement of modules.

The cells would be contained in each chamber with polycarbonate membranes (PC) (Ferrari et al., 2005; Bollmann et al., 2007; Kim et al., 2008; Nichols et al., 2010) of pore size dependent on the cultured microbial species, that would allow the exchange of small molecules by diffusion. The fluxes of media and intermediates between chambers would be controlled by valves and pumps made of PDMS as described by the work of Unger (2000), which offer many advantages such as having a short response time (1ms), being able to precisely control their opening (almost linear response to the applied pressure) and open/close perfectly up to rates of 75 Hz even in the presence of particulates, amongst others. Moreover, a gentle peristaltic pump (94% survival rate with *E. coli*) can be constructed by aligning several of these valves and actuating them sequentially.

#### 2.3.2. Sequential bioreactors

This concept would work similarly as sequencing batch reactors (SBR) used in wastewater treatment, where communicating sequential batches each fulfill a specific function (Robert and Arthur, 1979; Poltak, 2005). Hollow-fiber modules (Manjarrez et al., 2000) could connect well-stirred bioreactors (glass, polymer, stainless stell,..) that would each contain one or multiple microbial species accomplishing part of biochemical transformation. The flow of media and intermediate products between bioreactors could be controlled by applying pressure into the headspace of each vessel (Manjarrez et al., 2000) or using pumps (e.g., peristaltic,..) (Doran, 2012; Stanbury et al., 2017). Additionally, the cells could be even further confined and separated from the bulk of the medium by cellulose dialyzing membranes (Gerhardt and Gallup, 1963; Aida and Yamaguchi, 1966; Pörtner and Märkl, 1998; Ohno et al., 1999), or in dialysis tubing (Baker and Herson, 1978; Turley and Lochte, 1985; Gehin et al., 1996; Guedon et al., 1999), and those “sausages” could be placed inside each bioreactor.

To bridge the gap between the micrometer scale (nL to μL) of microfluidic chips and the lab-scale (100 mL to L) or even industrial size bioreactors (L to hL), perfusion bioreactors (mL to 100 mL) culturing cells in hollow-fiber cartridges could also be used (Li et al., 2009; Whitford and Cadwell, 2009; Bonham-Carter and Shevitz, 2011; Langer, 2011; Shevitz et al., 2011; Fraser and Endres, 2013; Langer and Rader, 2014). Besides being cheap and easily scalable, those hollow-fiber cartridges offer the modularity required for SLMC.

### 2.4. Strain selection of consortia members

As mentioned previously, this study focuses on engineering the environment in which the microbial consortium will be cultured, rather than engineering the microorganisms for a specific purpose, despite the many possibilities that synthetic biology offers (Hays et al., 2015). We will therefore mostly limit the discussion to the selection of the most appropriate microbial strains for the desired application, while not rejecting the idea of including genetically engineered organisms in our consortia, if those proved to be the best suited to fulfill a given function.

While assembling a microbial consortium, after choosing a species to perform a step of a biochemical process, the selection of the optimal strain of a species remains an open question. Considerations such as growth characteristics, yields, nutritional requirements, secreted by-products (detrimental or not to downstream microorganisms), resistance/resilience, would all play a role in the selection of a strain. If the goal is to produce biomass, it would make sense to choose the strain with the highest possible growth rate. In case of a chemostat where cells are flushed out in the effluent, a higher growth rate would allow a higher flow rate and therefore a higher production rate. Should the cells need to be contained in their compartments, without the possibility to control their population by flushing some of them out in the effluent, low growth rates might be desired or using methods to control their number in order to maintain their population steady. This could be achieved by periodically exposing part of the population to UV light, by limiting the availability of a nutrient to reduce growth, or with quorum-sensing methods (You et al., 2004; Wang et al., 2014). If several strains of a given species could fulfill the same function but had different yields, we would of course select the strain with the highest yield (gram product per gram substrate). The selection of the appropriate strain should also aim at reducing or even avoid the secretion of metabolic by-products that could inhibit downstream organisms (e.g., acetate, lactate, ethanol,..). Similarly, due to the ease by which nutrients could be added in line, it would be advantageous to prefer upstream strains with simple nutritional requirements (in trace elements, salts,..), to limit interferences with the metabolism of downstream microorganisms. Another important consideration is the resistance and resilience of the microorganisms, i.e., their ability to withstand environmental perturbations such as abrupt changes in nutrients concentrations, or recover readily after the disturbance occurred. Therefore, those characteristics would also have to be pondered for the selection of the appropriate member.

### 2.5. Considerations of community stability and renewal

Community stability is a desired feature of a microbial consortium for industrial applications. As SLMC will ideally grow the members of microbial consortium separately, as interacting monocultures, the risk for horizontal gene transfer and loss of engineered function should essentially be null. To limit the impact of evolution and the appearance of mutations that could give rise to “cheaters” (Johns et al., 2016) or other mutants, thereby reducing the performance of a consortium, we suggest replacing the cells with fresh ones, after a sufficiently short period of time. Solutions designed to reduce the evolvability of biological systems by increasing their genetic reliability have also been suggested and are described by Renda et al. (2014), but as mentioned previously, despite being compatible with SLMC, genetic engineering will not be the focus of this study. Alternatively, to reduce the impact of such an adverse event, we propose introducing redundancies at sensitive points prone to failure, in the form of multiple identical modules connected in parallel to provide an additional safety mechanism and improve the functional stability of the system.

Besides the genetic stability of a consortium discussed above, the robustness/stability of the food-chain or food-web a consortium represents is also of paramount importance. Changes in environmental conditions (nutrients, temperature, pH,..) could lead to strong fluctuations in microbial populations which could have disastrous consequences for downstream populations relying on metabolites produced by upstream microorganisms. Once more, the tuning of the operating conditions will determine how perturbations propagate through the system while trying to reduce those to a minimum.

### 2.6. Potential applications

In addition to potentially improving the productivity of current co-cultures by providing more suitable growth conditions to each member of a microbial consortium, SLMC would dramatically expand the range of applications by allowing the combination of microorganisms that typically are not found in natural systems due to incompatibility of environmental conditions (or occur very rarely in nature; Table 1).

**Table 1:** Potential applications.

**Application**	**Product/process**	**Microorganisms**	**Environmental requirements**	**Platform, scale**	**References**
Nitrogen removal	Conversion of ammonium to nitrogen gas (Anammox)	*Nitrosomonas* sp. and *Candidatus* Brocadia anammoxidans	Aerobic and anaerobic	Bioreactors	Strous et al., 1997; Jetten et al., 2001; van Dongen et al., 2001; Devol, 2003; Kuypers et al., 2003; Arrigo, 2005; Dalsgaard et al., 2005; Kuenen, 2008; Kartal et al., 2010
Bioremediation	Degradation of hydrocarbons	*Geobacter metallireducens* and oxygen consuming microbial species	Anaerobic conditions for degrader	Alginate beads	Lovley et al., 1993; Butler et al., 2007
Pharmaceuticals	Stepwise assembly of biologically active proteins	Genetically engineered *E. coli, S. cerevisiae*,..	Some processes might require different pHs, temperatures,..	Microfluidics, bioreactors	Wacker et al., 2002; Ihssen et al., 2010; Kamionka, 2011; Rosenberg et al., 2013; Baeshen et al., 2014, 2015
Biofuels	Bioconversion of lignocellulosic material into bioethanol and biodiesel	*Clostridium thermocellum, Zymomonas mobilis, Pichia stipidis* and *Acinetobacter baylyi*	Different temperature and oxygen requirements	Industrial size bioreactors	Zaldivar et al., 2001; Kalscheuer, 2006; Fu et al., 2009; Maki et al., 2009; Zuroff and Curtis, 2012; Lin et al., 2013
Space missions	Complete recycling of waste (closed-loop)	e.g., Thermophilic Anaerobic Bacteria, Nitrifying Bacteria, Photoautotrophic Bacteria,..	Anaerobic, aerobic, light,..	Microfluidics, bioreactors	Gòdia et al., 2002; Hendrickx et al., 2006; Lasseur et al., 2010; Menezes et al., 2014

*Potential areas of application for SLMC, the biochemical process, the organisms involved and their environmental requirements*.

#### 2.6.1. Nitrogen removal

**Figure 3:**
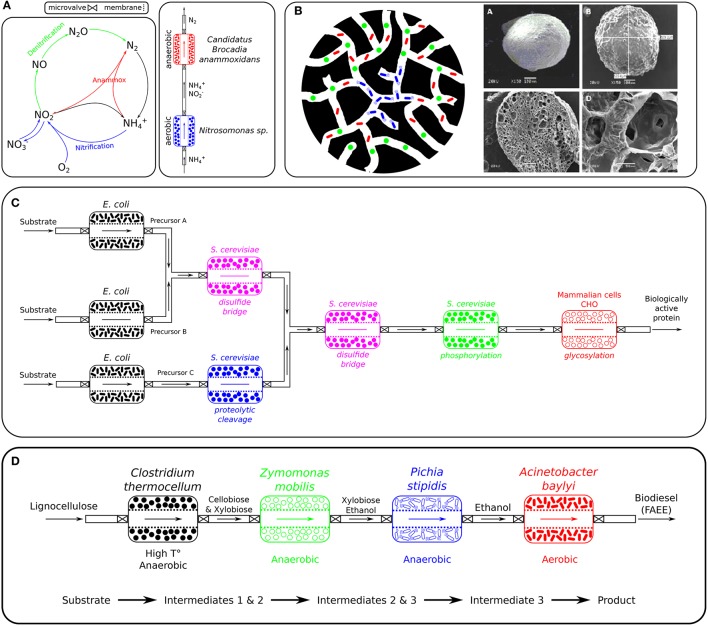
Potential applications of SLMC. **(A)** Nitrogen removal: Nitrogen cycle and suggested layout of the consortium for direct conversion of ammonium to nitrogen gas. This bioprocess requires the conversion of part of the ammonium to nitrite under aerobic conditions (here by *Nitrosomonas* sp.) before the anaerobic ammonium oxidation (anammox), here performed by *Candidatus* Brocadia anammoxidans, can take place and combine ammonium (NH_4_) and nitrite (NO_2_) to nitrogen gas (N_2_). **(B)** Bioremediation: Porous alginate microbeads containing a microbial consortium. The left illustration represents a porous bead and an embedded microbial consortium degrading a specific pollutant. The microorganism responsible for the degradation of the pollutant (blue: e.g., *Geobacter metallireducens*) could be placed in the core of the porous bead, while the metabolic activities of microbes surrounding it provide the necessary anoxic conditions (green: oxygen consumer) and nutrients (red: by-products feed the blue member) the degrader requires to fulfil its catabolic function. Alternatively, all three microbes could each perform one step of a three steps biodegradation process. The right scans (electron microscope) show alginate beads, a cross-section and the porous network of such a bead. **(C)** Pharmaceuticals: Pharmaceutical application of modular microbial consortia. *E. coli* would generate the basic components as they are a prolific organisms that is easy to engineer and can produce high yields while being cost effective. As *E. coli* mostly lacks the ability to perform posttranslational modifications and since those are vital for the biological activity of human proteins, other systems such as yeasts (*Saccharomyces cerevisiae, Pichia pastoris*,..) or mammalian cells (CHO,..) could be used to modify those building blocks and produce the biologically active protein. **(D)** Biofuels: Biodiesel production from lignocellulosic material. *Clostridium thermocellum* would first break down lignocellulose into 5- and 6-carbon sugars at high temperature and anaerobic condition, that would then be fermented by *Zymomonas mobilis* and *Pichia stipidis* to ethanol. Finally, the strict aerobe *Acinetobacter baylyi* would convert ethanol to biodiesel. Image source **(B)** Soliman et al. (2013).

Anaerobic ammonium oxidation (Anammox) is an important process in the marine nitrogen cycle that converts ammonium ($NH_{4}^{+}$) and nitrite ($NO_{2}^{-}$) under strictly anaerobic conditions, directly to nitrogen gas (N_2_), and is believed to be responsible for 30–50% of the N_2_ gas produced in the oceans (Devol, 2003; Kuypers et al., 2003; Arrigo, 2005; Dalsgaard et al., 2005). Before anammox bacteria, such as members of the Planctomycetes (e.g., *Candidatus* Brocadia anammoxidans, *Candidatus* Kuenenia stuttgartiensis,..), can combine ammonium and nitrite to nitrogen gas (N_2_), ammonia oxidizing bacteria (e.g., *Nitrosomonas*) need to convert part of the ammonium to nitrite under aerobic conditions (Kuenen, 2008). A current application of the anammox process is in wastewater treatment. By replacing the denitrification step (Figure 3A) completely, and saving half of the nitrification aeration costs, it reduces operational costs by 90% (Strous et al., 1997; Jetten et al., 2001; van Dongen et al., 2001; Kartal et al., 2010).

The compartmentalization strategy offered by the modularity of SLMC would surely benefit such a process which requires the combination of incompatible biochemical reactions (aerobic/anaerobic). *Nitrosomonas* would be grown in a first module under oxic conditions to convert part of the ammonium to nitrite. The ammonium/nitrite mixture could then be transferred to a second niche culturing *Candidatus* Brocadia anammoxidans which would combine $NH_{4}^{+}$ and $NO_{2}^{-}$ to N_2_ in an anoxic environment (Figure 3A).

#### 2.6.2. Bioremediation

One potential application for SLMC is bioremediation as it has been shown that microbial consortia perform better at degrading certain pollutants than single species (Boonchan et al., 2000; Kim and Lee, 2007). Bioremediation is a method that uses microbial activity to destroy toxic pollutants. It offers a good alternative to conventional remediation methods such as incineration, burying, solidification and thermal desorption and some of its advantages are its low-cost, low-technology techniques, it can often be carried out on site, is environmentally safe and does not generate waste (McDonald, 1993; Vidali, 2001; Lovley, 2003; Robles-González et al., 2008; Wijffels, 2015).

*Ex situ* methods such as slurry or aqueous bioreactors are more controllable and often result in higher degradation rates, but are also more expensive than *in situ* bioremediation, and disrupt the environment when the soil is excavated or the groundwater is pumped out of the soil before treatment (Vidali, 2001; Robles-González et al., 2008). Sequential bioreactors as suggested by SLMC would offer a good platform to perform such treatments. As the degradation of the pollutant is often a multistep process, the different microbial species composing the degrading consortium would be cultured sequentially, following those degradation steps. Once more, the spatial separation of processes offered by SLMC would allow for a higher controllability than the mixed-cultures currently used (Vidali, 2001; Robles-González et al., 2008).

*In situ* bioremediation offers a gentle alternative to environmental remediation as the microorganisms responsible for the degradation of the pollutant(s) are “inserted” into the environment (bioaugmentation). In such a context, as the environmental conditions are not controlled, one needs to ensure that those would be suitable for the microbial consortium in terms of temperature, pH, nutrients,.. A suggested solution to control the spatial distribution and influence the environmental condition of the degrading consortium would be the construction of porous alginate beads (Lee and Mooney, 2012) containing the microbial consortium (Haferburg and Kothe, 2010) and dispersing them in the contaminated environment (soil, groundwater,..). It has been shown that packing microbes in such beads does not impact the survival nor the performance of the microorganisms, although one limitation of such constructs might be a reduced bioavailability of the pollutants to the consortium (Scherer et al., 1981; Klein et al., 1983; Luthy et al., 1997; Lee and Heo, 2000).

The characteristics of the beads (physical properties, nutrient composition,..) would be tailored to the microbial consortium. Nutrients and pollutants to be degraded would be transported by diffusion to the degraders trapped inside the beads, which will limit our control over the system. *Geobacter metallireducens*, for example, is capable of coupling the degradation hydrocarbons and monoaromatic compounds with the reduction of Fe(III), Mn(IV), U(VI) and other heavy metals, but is a strict anaerobe (Lovley et al., 1993; Butler et al., 2007). Combining it with an oxygen consuming microorganism would create the anoxic environment it requires to perform its biodegrading activity (Figure 3B). An additional consortium member could also support the degrader by converting a substrate present in the local environment into a metabolite required by the degrader. As alginate is biodegradable (Aggarwal et al., 1999; Ueng et al., 2007), should those microorganisms survive in their new environment upon their release from the beads (which rarely occurs, Vidali, 2001), one would still need to consider the ecological consequences of inserting exogenous microbial species in that ecosystem (Tiedje et al., 1989; Vidali, 2001; Robles-González et al., 2008; Wijffels, 2015).

#### 2.6.3. Pharmaceuticals

The pharmaceutical industry heavily relies on microorganisms for the production of drugs, such as antibiotics, antitumor agents, immunomodulators, enzyme inhibitors, antiprotozoal agents, nematicides, and insecticides. The most important hosts for the production of pharmaceutical recombinant proteins are the bacterium *Escherichia coli*, the yeasts *Saccharomyces cerevisiae* and *Pichia pastoris*, and mammalian cell lines like Chinese hamster ovary cells (CHO). Some of the advantages of using *E. coli* are the ease of engineering their genome, their rapid growth, easy culture and high product yields. One major limitation of *E. coli* or unicellular bacteria in general when it comes to producing eukaryotic recombinant polypeptides, is that they lack the ability to perform the posttranslational modifications necessary for the biological activity of some proteins, most importantly glycosylation. This issue was partially overcome by transferring the ability of the bacterium *Campylobacter jejuni* to glycosylate proteins to *E. coli*, although the structure of the glycosylation differs from that observed in eukaryotes (Wacker et al., 2002; Ihssen et al., 2010).

Yeast, like *Saccharomyces cerevisiae*, is often the preferred host for expression of proteins that require posttranslational modification for its biological activity. Yeast cells can carry out many posttranslational modifications, such as phosphorylation, glycosylation, acetylation and acylation, and express recombinant proteins in a soluble and properly folded active form. One major concern for the production of glycosylated proteins is that the glycosylation performed by yeast confers the protein with a short half-life *in vivo* and generates hyper-immunogenicity which makes the proteins less effective (Rosenberg et al., 2013; Baeshen et al., 2014). Mammalian cells (e.g., CHO) are usually the best expression systems for proteins which cannot be properly posttranstionally modified by bacteria, but their main drawbacks are their poor secretion, which complicates the purification process, and results in high production costs (Kamionka, 2011; Rosenberg et al., 2013; Baeshen et al., 2014, 2015). The need to produce human proteins with the proper posttranslational modifications for their activity, using a combination of microorganisms rather than a single engineered species could provide an answer to the drawbacks mentioned above. Each microbial species used would be specialized in a single step of the production of the protein. One or multiple microorganisms could produce precursors or parts of the proteins, which would be assembled (e.g., disulfide bridges) and modified downstream (e.g., glycosylation, methylation, phosphorylation, proteolytic cleavage,..), using at each step the organism that performs better at that specific task (Figure 3C).

#### 2.6.4. Biofuels

The production of biofuels from lignocellulose could also be an area where the structure provided by SLMC could facilitate the biochemistry of the microbial species involved (Zuroff and Curtis, 2012). Besides being the most abundant raw material on earth, lignocellulose offers the advantage of being a renewal source of energy. It is composed of carbohydrate polymers (cellulose, hemicellulose), and an aromatic polymer (lignin). These carbohydrate polymers contain different sugar monomers (six and five carbon sugars) and it is difficult to engineer a single organism capable of the simultaneous degradation of both sugars do to a preference for glucose (Zaldivar et al., 2001). Therefore, a consortium composed of one organism only capable of metabolizing hexose while the other consumes pentose, was shown to be more suited for the fermentation of such a substrate (Fu et al., 2009). In this study, a combination of the bacterium *Zymomonas mobilis* and the yeast *Pichia stipitis* was used and although they achieved yields of more than 96% of the theoretical value, they noticed an inhibitory interaction of *Zymomonas mobilis* on *Pichia stipitis* when grown in co-culture. Growing each species separately would allow to provide both species with more suitable growth conditions (nutrients, T°, pH,..) and avoid this inhibitory interaction, while making the bioprocess continuous.

In order to extract from raw plant material (lignocellulose), the 6-, and 5-carbon sugars that *Zymomonas mobilis* and *Pichia stipidis* respectively need, the catabolic activity of the thermophilic anaerobic bacterium *Clostridium thermocellum* could be used. The cellulase and hemicellulase it produces break down lignocellulose into the disaccharides cellobiose and xylobiose that could then be fermented into ethanol downstream (Maki et al., 2009). Since this step requires a higher temperature than the following fermentative steps, separation from processes would also be beneficial here.

Furthermore, due to its modularity, SLMC would allow us to attach additional bioprocesses downstream of the ethanol producing consortium in order to convert ethanol, for example, into biodiesel (Lin et al., 2013). Besides an urgent need to transition the world's fuel production from fossil to renewable fuels, biodiesel offers several advantages over petroleum-based diesel, such as being completely biodegradable, non-toxic and reducing emissions of carbon monoxide, sulphur, aromatic hydrocarbons and soot particles (Kalscheuer, 2006; Lin et al., 2013). While the lignocellulose conversion into sugars followed by their fermentation to ethanol are strictly anaerobic processes, its further transformation into biodiesel requires an oxic environment. The addition of a separate bioprocess is therefore necessary in order to use a bacterium such as *Acinetobacter baylyi* (strict aerobe) for the conversion of bioethanol into biodiesel (Kalscheuer, 2006) (Figure 3D).

#### 2.6.5. Life support systems for deep space exploration

A promising area of potential application for SLMC could be space mission and the development of life support systems for long distance space exploration, such as the Next Generation Life Support project (NGLS) from NASA and MELiSSA (Micro-Ecological Life Support System Alternative) which is a current project from the European Space Agency (ESA). One objective is to create a complete recycling system for gas, liquid and solid wastes using the combined activities of different microorganisms, plants as well as the human crew (Gòdia et al., 2002; Hendrickx et al., 2006; Lasseur et al., 2010; Maggi and Pallud, 2010a,b; Jones et al., 2012; Barta et al., 2014). While they suggest using single strains or mixed cultures (depending on the bioprocess), we believe that the structured microbial consortia approach offered by SLMC would provide the controllability, predictability and stability necessary for such applications. Another objective of NASA's NGLS project for long duration manned missions to explore the Moon and Mars is to utilize local raw materials (*in situ* resource utilization) in order to reduce the payload necessary to launch (Menezes et al., 2014). Their idea is to employ microorganisms to produce propellant, food, biopolymers and pharmaceuticals, by using as much as possible resources found locally (i.e., Moon or Mars). Whereas those studies consider synthetic biology to engineer single species for each product, we recommend a microbial consortia approach, for the many reasons mentioned in the introduction that make consortia more interesting for bioprocesses (can accomplish more complex tasks, improved bioconversion efficiency, modularity,..). As the reliability of such systems is vital for those missions, we believe that our platform could provide the structure necessary for those consortia to exhibit the controllability and stability required for such applications.

An additional potential application addresses unculturable microbial species. Only 7,000 bacterial species have been validly described out of an estimated bacterial diversity upward of one trillion (10^12^) microbial species and most likely orders of magnitude more unique compounds with potentially valuable functions such as novel antibiotics (Davies, 2007; Sabra et al., 2010; Stewart, 2012; Locey and Lennon, 2016). It is suggested that two main reasons are linked to our inability to culture most microorganisms, one being the difficulty to reproduce the environment in which these species grow and the second being the lack of knowledge regarding the interactions between those species (Stewart, 2012). With better understanding of the environmental conditions required as well as the identity of the interacting microorganisms, we believe that the modularity and controllability of SLMC would allow us to reproduce suitable conditions more easily than existing culture platforms and finally be able to grow those unculturable species and harvest the potential benefits of their metabolisms.

Besides the industrial applications and engineering aspects mentioned above, SLMC represents an ideal research tool to study interactions between microorganisms in a quantitative and systematic way. The content of one or multiple growth chambers, each containing monocultures, could for example be fed to a single microorganism or a consortium to test how it impacts its growth. By avoiding the co-culture of the microbial species involved in the experiment, we would more easily be able to control the population size of the different members and prevent the extinction of one or more species, thus improving the functional stability of the consortium. In the following we will present the results of a study as well as a mathematical model we developed to corroborate the claims we made so far in support of SLMC.

## 3. Yield increase by segregation of processes

**Figure 4:**
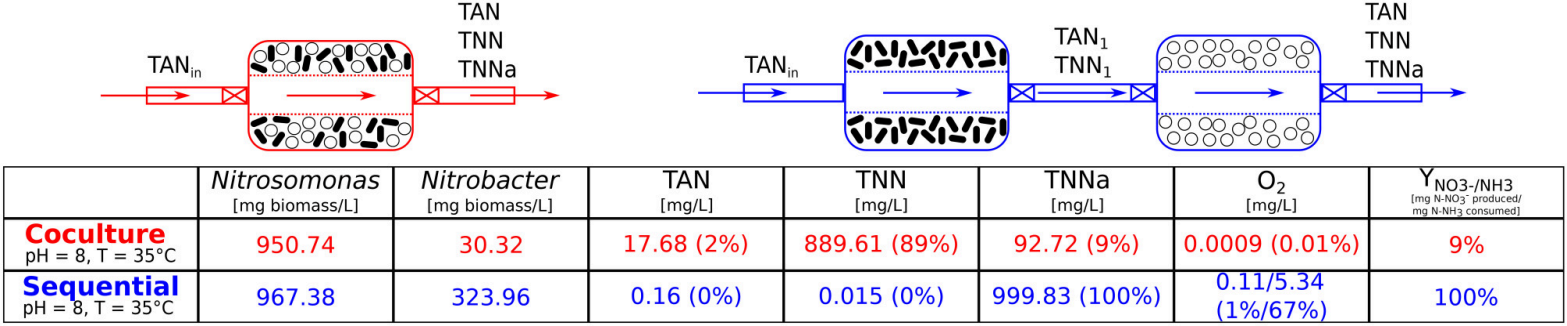
Co-culture vs. Sequential: steady-state values. This illustration shows the two layouts of bioreactors that are compared for their ability to convert ammonia to nitrate. In the co-culture (red) both microbial species are cultured together, whereas in the sequential scenario they grow in separate bioreactors. The first chamber contains *Nitrosomonas* sp. which convert ammonia (*NH*_3_) to nitrite ($NO_{2}^{-}$) and its content is fed to the second bioreactor culturing *Nitrobacter* sp. which oxidizes nitrite to nitrate ($NO_{3}^{-}$). The steady-state values of the microbial populations, nitrogen species (total ammonium nitrogen, TAN $=N-NH_{3}+N-NH_{4}^{+}$, total nitrite nitrogen, TNN $=N-HNO_{2}+N-NO_{2}^{-}$ and total nitrate nitrogen, TNNa $=N-HNO_{3}+N-NO_{3}^{-}$) and oxygen concentration (first and second bioreactor values) are listed for the co-culture and the sequential bioreactors (same pH and temperature as in co-culture). Microbial populations are given in [mg biomass/L] and the chemical species in [mg/L]. The percentages indicated next to the values are relative to the total nitrogen injected into the system (TAN_in_) therefore the sum of all the nitrogen species is equal to the input concentration, here [TAN_in_] = 1, 000 [mg TAN/L]. The values for the nitrogen species indicated for the sequential scenario are the output concentrations of the second bioreactor (containing *Nitrobacter*).

The need for a quantitative assessment of the potential offered by SLMC motivated us to develop a simple temporal model, based on ordinary differential equations, with the purpose of testing two layouts of bioreactors to culture a microbial consortium of two microorganisms. In one scenario both species were simulated as a co-culture, which was compared for its performance with sequential bioreactors, each containing one of the members (monocultures) (Figure 4). Far from reflecting the degree of complexity that can be reached by SLMC, this model was only intended as a quantitative argument in support of our concept.

Due to the vast amount of literature available (Painter, 1970; Wiesmann, 1994; Agency, 2002; Prosser, 2005; Ward, 2008; Grady et al., 2011) we chose as model consortium the nitrifiers *Nitrosomonas* sp. and *Nitrobacter* sp. The two microbial species are involved in the nitrification process, part of the nitrogen cycle (Figure 3A) and are main actors for nitrogen removal in wastewater treatment (Henze et al., 1997; Dochain and Vanrolleghem, 2001; Metcalf & Eddy Inc. et al., 2003; Grady et al., 2011; van Haandel and van der Lubbe, 2012). In our simulations, and under identical operating conditions, the sequential bioreactors outperformed the co-culture in terms of yield, as can be seen in Figure 4. This was mostly due to a high competition for substrate and to a lesser extent to higher inhibiting effects observed in co-culture, which are similar observations made by Fu et al. (2009). Although SLMC allowed us to tune the environmental conditions by adapting the temperature and pH to optimal values for each microorganism, this did not translate into an increase in yield as the consortium already operated at the maximal yield (100% bioconversion). For further modeling details please refer to the Supplementary Material.

Fu et al. (2009) tested different fermentation schemes in order to improve the ethanol production of a consortium composed of the bacteria *Zymomonas mobilis* and the yeast *Pichia stipitis*, using a glucose/xylose mixture as carbon source. This work showed that a sequential approach, operationally similar to SLMC, resulted in a clear increase in yield and substrate consumption over a co-culture. Where in co-culture the sugar consumption reached 86–91% and the yield for ethanol production Y_P/S_ 0.38–0.43 [g EtOH/g sugar], other fermentation schemes relying on a temporal separation of processes achieved a complete sugar consumption (100%) and yields of 0.42–0.50 [g EtOH/g sugar], which corresponds to over 96% of the theoretical maximum (Fu et al., 2009).

They hypothesized that the lower yields and bioconversion efficiency observed in co-culture were due to the inhibiting effect of *Z.mobilis*' metabolism on the fermentative activity of *P.stipidis*, as well as competition for certain metabolites. Therefore, separation of both species is a prerequisite for a successful sugar mixture co-fermentation by these two strains. By spatially segregating both microorganisms and providing them with optimal growth conditions, SLMC would not only prevent the metabolic interferences observed in co-culture (inhibitions and competition for metabolites), but potentially further increase the yield of ethanol fermentation over the temporal fermentation schemes presented in this study.

## 4. Challenges and opportunities

Realizing the potential offered by the proposed SLMC would require overcoming challenges of different types (engineering, biological and operational) to ensure stability and functionality of target consortia. The increased controllability resulting from the compartmentalization of bioprocesses comes with some limitations and potential solutions are proposed in the following section to highlight the expected complexity of such systems. Clearly, the discussion remains general at this stage as many of the issues of initiation, stability, operation, and resetting would be tailored to the system of interest.

### 4.1. Initiation

Once the different members of the consortium are in place, how should a system be initiated in order to start production? For small scale platforms such as microfluidic chips, the transport and storage of pre-assembled microbial consortia, could be accomplished by freezing (Mazur, 1984), drying (Morgan et al., 2006) or lyophilization (Gitaitis, 1987) of microbial cells. The method would be based on the tolerance of the microorganisms involved to those preservation procedures.

In the case of dried or lyophilized cells, distilled water would be added into each module to rehydrate the cells and allow them to regain their full activity before starting the fluxes of media (Gitaitis, 1987). At the beginning, the system would go through a synchronization phase where the species contained in the different operational elements would adapt to the changing environmental conditions, until a stable phase or steady regime is reached. To limit the impact of the initial switch from distilled water to media, a smooth transition slowly increasing the concentration of the media could be performed to give the cells more time to acclimatize to their new environment.

### 4.2. Functional stability: redundance, microbial resistance and resilience

Functional redundancy (several species fulfilling the same function) as observed in nature amongst members of a microbial community, increases the stability of the community to environmental fluctuations (Allison and Martiny, 2008; Konopka, 2009; Konopka et al., 2015). In a similar way, the modularity inherent to SLMC would allow the introduction of redundancies (multiple functional units connected in parallel and containing the same microorganism) in particular if a step along a biochemical pathway was sensitive and prone to failure. In such a case the system would continue to operate even if one of the operational elements failed and needed to be reset. Those redundant modules would reduce the impact of an adverse event on the system (e.g., mutation, contamination,..) and would ensure that every building block necessary would be available at every step of the biotransformation, therefore improving the stability of the bioprocess. This would be especially important for applications such as life support systems for space exploration where functional stability is of paramount importance (Gòdia et al., 2002; Hendrickx et al., 2006; Lasseur et al., 2010; Menezes et al., 2014).

As the different species would ideally be grown as monocultures to offer the highest level of controllability, it will also be important to take into account their resistance and resilience to perturbations while designing a consortium (Allison and Martiny, 2008). The active population size of sensitive members could experience large variations and the associated fluxes of by-products for downstream requirements could trigger a cascade of instability in the system. Therefore, should several species perform the same biochemical process of interest, one could select the microorganism that is the most robust to environmental fluctuations, i.e., the most resistant and/or resilient [e.g., *Nitrosomonas, Nitrospira* and *Nitrosococcus* are capable of oxidizing ammonia (Ward, 1996; Zehr and Kudela, 2011; Hatzenpichler, 2012), and *Nitrobacter, Nitrospina* and *Nitrococcus* are nitrite oxidizers (Nowka et al., 2015; Koops, 2001)]. An alternative would be to adapt the operating conditions to fit the tolerance for perturbations of the most sensitive member.

### 4.3. Operating conditions and control elements

The purpose of tuning the operating conditions (fluxes, temperature, pH, DO,..) is to maximize the function of the consortium (product concentration, biomass production,..) while considering the resistance and resilience of the most sensitive microorganism. The possibility of operating a system using continuous or prescribed pulses of media offer various options to steer the system. With a continuous flow, the main control element is the dilution rate D, which depends on the flux Q of injected liquid and the volume V of the operational element (D = Q/V). By reducing the dilution rate we reduce the amount of media that enters a module per time unit and therefore the rate of change of concentrations inside that functional unit. Furthermore, the resulting increase in residence time τ for media in each module (τ = 1/D) would give the cells more time to consume the substrates supplied by the media. Finally, with a continuous flow, most of the perturbations would occur during the initiation phase, until a steady-state is reached (under constant input concentrations and environmental conditions), and the level of fluctuations would therefore be limited.

**Figure 5:**
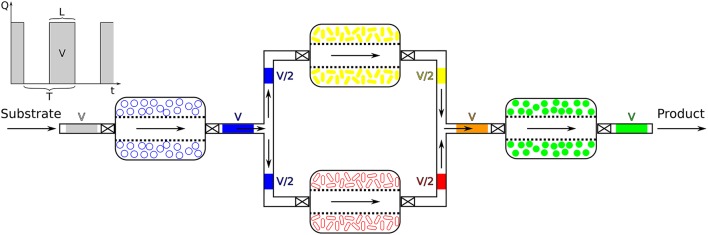
Pulsing mode. The height of the pulses represents the flux Q of media into an operational unit. The period T and pulse length L determine the duty cycle (L/T), which is the percentage of one period during which the pulse is “ON”. The volume V injected with each pulse is V = Q^*^L.

Conversely, a pulsing regime would generate periodic perturbations, and considerations of microbial member robustness and efficiency under fluctuating concentrations become very important. An advantage of the pulsing mode, is that it offers a more subtle control than a continuous flow, as the volume of media injected at each cycle (of period T) depends on the flux Q and the duration L of the pulse (Figure 5). Rather than with the constant flux of the continuous flow, the same volume could be injected in a bioreactor with a short pulse L and a high flux Q over the same period T and give the cells a “resting time” between pulses, albeit at the expense of higher fluctuations. This resting time would allow the cells to accomplish their function, before receiving the next “load” of substrate(s) to convert. The pulses could also take the form of a trigonometric function (sine, cosine) to offer smoother transitions to the cells. The period T and the pulse length L could also be varied, for example to better accommodate the requirements of a growing cell population until it reaches a quasi-steady-state. However, we do not believe that variations in concentrations would be a main issue, as one benefit of using sequential growth modules as proposed by SLMC comes from the transfer of media from one element to the next which has a dampening effect on downstream concentration fluctuations. This dampening is proportional to the dilution rate. The smaller the dilution rate, the smaller the effect upstream fluctuations will have on changes in concentrations in downstream modules.

The core control of the proposed SLMC lies in the capability of tuning spatially segregated environmental conditions for each member of the consortium (or group of members), which allow us to provide the microbial species with optimal growth condition. Physical and chemical aspects of the environment could be adjusted by controlling for example the pH, temperature and dissolved oxygen (DO) for each species without interfering with the rest of the system. The pH could be regulated by adding, acid or base in-line (Keen and Prosser, 1987; Wijffels et al., 1991; Hunik et al., 1994; Gernaey et al., 1998; Carvallo et al., 2002; Park and Bae, 2009), a buffer to the medium (Shieh and LaMotta, 1979; Charley et al., 1980; Hellinga et al., 1999; Chandran and Smets, 2005; Vadivelu et al., 2006), or by inserting a “buffering chamber” between two elements, an abiotic module whose function would be to adapt the pH of the medium before piping it to the next microorganism, should this be necessary. Temperature of bioreactors could be controlled by placing it in a thermostatically controlled bath, by using internal heating coils or external heating jackets (Stanbury et al., 2017). For the microfluidic scale, external temperature control methods relying on thermoelectric effects (Peltier elements) or resistive heating (Joule heating) exist (Miralles et al., 2013). To avoid applying a thermal source, alternatives based on electromagnetic radiation (microwaves, laser,..) destined at directly heating the bulk of the liquid could also be employed (Miralles et al., 2013). The choice of the heating technique would be determined by the tolerance to those methods of the microbial species composing the consortium. If the dissolved oxygen requirements of two consecutive microbial species were incompatible, several techniques to reduce the oxygen concentration in the liquid phase exist. Depending on the scale, sonication in addition to reduced pressure, membrane degasification, substitution by an inert gas such as nitrogen (sparging) or addition of a reductant like sulfite salts (e.g., sodium or ammonium sulfite) which react with oxygen to form sulfate ions, could remove excess dissolved oxygen (Butler, 1994; Wiesler and Sodaro, 1996; Degenhardt et al., 2004; Wang, 2006). Should the dissolved oxygen level need to be increased, local influx of air/oxygen could be provided separately. In the case of well-mixed bioreactors, the stirring would offer an additional control element as characteristics such as the stirrer speed and the impeller geometry would impact the concentration of dissolved oxygen (Garcia-Ochoa and Gomez, 2009; Doran, 2012; Stanbury et al., 2017).

The medium would also offer some flexibility as achieving a medium composition capable of maintaining multiple species would not be necessary since most modules would ideally contain monocultures. Compounds required by a specific species could be supplied along the way, at any given step of a biochemical process, as long as they would not be detrimental to “downstream” organisms or reach inhibiting concentrations in the effluent, irrelevant of the effect they would have had in co-culture, on “upstream” organisms. This highlights the previously mentioned decoupling offered by SLMC as upstream elements are independent from downstream sections.

Controlling the microbial population size is another tool we can leverage. In nature, should a member of a microbial community fulfil an essential function for the survival of the community (release an enzyme catabolizing the production of substrates or the degradation of a toxin or antibiotic), but appear in small numbers due, for example to competition for nutrients, it could create a bottleneck limiting the growth of that community and therefore its ability to perform its function. By controlling the growth conditions of each microbial population separately, the population of that species could be increased to remove the bottleneck it represents. Conversely, if microbial growth needed to be limited or the population reduced, substrate limitation to limit growth or a physical treatment such as exposing parts of the microbial population to UV light to inactivate some of the cells could be envisioned (Chang et al., 1985; Rowan et al., 1999; Hijnen et al., 2006). Additionally, as any environmental change would mostly impact a single species rather than the whole community, and thus avoid the complex population dynamics that would occur in a co-culture, homeostasis could be more readily achieved.

### 4.4. Resetting a module after an adverse event

When culturing different microbial species in separate but connected functional units, external contamination as much as internal cross-contamination is a major concern. Several methods of cellular confinement, such as polycarbonate membranes (PC) (Ferrari et al., 2005; Bollmann et al., 2007; Kim et al., 2008; Nichols et al., 2010), dialysis tubing (Baker and Herson, 1978; Turley and Lochte, 1985; Gehin et al., 1996; Guedon et al., 1999), hollow fiber bridges (Manjarrez et al., 2000) and cellulose dialyzing membranes (Gerhardt and Gallup, 1963; Aida and Yamaguchi, 1966; Pörtner and Märkl, 1998; Ohno et al., 1999) have been discussed previously, and could be used to isolate each microbial species to its operational element, preventing such events, while also avoiding interspecies horizontal gene transfer.

Should the monitoring of a module (Pohlscheidt et al., 2013) reveal that an adverse event occurred (e.g., mutation, contamination,..), this would require the inactivation of the faulty unit, followed by its resetting/replacement. For microfluidics, using a physical method such as UV light exposure, would not only allow to terminate the cultured member of the consortium, as well as potential contaminating microorganisms (bacterial spores, viruses, amoebic cysts,..), but it provides a better alternative than chemical methods as it does not produce undesirable by-products (Chang et al., 1985; Rowan et al., 1999; Hijnen et al., 2006). The sterilized content would then be flushed out before replenishing the local niche with “fresh” cells. Alternative methods of sterilization rely on one or a combination of physical (autoclaving, UV light) and chemical treatments (ethanol, ethylene oxide, hydrogen peroxide,..) (Skaalure, 2008; Horst et al., 2009; Zhang et al., 2014; Yavuz et al., 2016), and could be applied to SLMC, for larger scales (e.g., bioreactors,..), depending on the tolerance of the materials used. Ultimately, in order to limit the risk of the occurrence of adverse events, we suggest the periodic replacement of the cells. If this information was not available, measurements of mutation frequencies for the selected media and operating conditions could be performed to determine the frequency of the replacements (Drake et al., 1998; Rosche and Foster, 2000; Foster, 2006).

## 5. Discussion and outlook

Tests of the proposed concepts of spatially linked microbial consortia would invariably involve a set of experiments to provide “proof of concept” of key elements of the SLMC, that under prescribed conditions the SLMC would support assembly of incompatible members or would outperform mixed communities. The simplest consortium (i.e., two interacting species) would be an ideal starting point as it would limit the complexity of the system and allow us to gain the experience necessary to construct more complex consortia. Experiments designed to evaluate the functionality, stability and efficiency of the chosen consortium in co-culture and in sequential modules will be conducted. In an initial phase, we would run both scenarios under the same conditions, providing the cultured cells with identical substrate composition, temperature, pH and, for aerobes, dissolved oxygen concentration. Microbial populations and concentrations of substrate, intermediate and product could be measured in the effluent to determine the yield of the microbial consortium for each scenario. In a second phase, we would capitalize on the separation of processes in the sequential scenario, and tune the conditions (temperature, pH,..) in each module to optimize the function of both microbial species. Here, once more, the yield would be determined, as a measure of productivity/efficiency, to compare it with the previous phase.

With the development of the skills for culturing different interacting microbial species residing in different (but interlinked operational units), we envision tests of assembling naturally incompatible consortia such as aerobe/anaerobe, thermophile/mesophile, acidophile/neutrophile/alkaliphile, or any combination of those. Theoretically, there would not be an upper limit to the complexity of the consortia that could be constructed and we could easily envision assembling microbial consortia ranging from two members to “super-consortia” composed of multiple interacting “sub-consortia” (Figure 1D). Such constructs would be made possible by the modularity inherent to SLMC, which would facilitate the assembly of microbial consortia of increasing complexity by the addition of extra modules. One could imagine developing a library of microbial consortia that could easily be connected in a plug-and-play manner, very much like words are assembled to form sentences.

To guide the design of our microbial consortia and experiments, we intend to use a flux balance analysis (FBA) approach (Orth et al., 2010, 2011; Krömer et al., 2014), with additional thermodynamic constraints (Henry et al., 2007; Schellenberger et al., 2011; Soh and Hatzimanikatis, 2014), to help us determine potential combinations of microorganisms based on their metabolisms as it would allow us to link the genetic information about the members and the environmental conditions (substrates, temperature, pH,..) to the substrate consumption, the growth rate and the production of the compound(s) of interest, to predict and optimize the desired function. Depending on the experimental platform used, especially if diffusion plays an important role, combining FBA and individual based modeling (IBM) would encompass the spatial information necessary to accurately simulate such processes (DeAngelis and Gross, 1992; Kreft et al., 1998, 2001; Grimm and Railsback, 2005; Lardon et al., 2011; DeAngelis and Grimm, 2014).

## 6. Conclusion

Microbial consortia require specific environmental conditions and spatial structure in order to interact and accomplish the biochemical processes they catalyze. Current applications of microbial consortia lack the necessary diversity in environmental conditions that is required for many biotransformations. Owing to its structure and modularity, SLMC capitalizes on the advantages of working with microbial consortia and the immense degrees of freedom to connect and enforce interactions between its members, whether or not they co-exist or interact in nature. This will not only enable the construction of novel microbial consortia and improve the controllability, predictability and stability over existing culture methods, but also potentially increase the productivity/yield of natural consortia.

Some of the hurdles that SLMC faces are of an engineering nature, such as the maintenance of a stable and functional system composed of metabolically interdependent members that should function at capacity and carry out the intended functions at the prescribed rates. Other challenges concern the monitoring and identification of the state of keystone elements in the system, to assess the performance of the consortia. SLMC will borrow concepts from natural ecological interactions and will expand into the uncharted area of synthetic microbial ecology, where the environments and the interactions among consortium members will be optimized. Various engineering solutions already exist to pursue simple prototypes and test the concepts by combining modeling and simple laboratory setups. The advancement of high dimensional FBA and thermodynamic contraints would make the selection of members and tuning the parameters to favor desired metabolic pathways feasible in the foreseeable future. The potential of engineering new interacting communities within the framework of synthetic ecology opens the door to harnessing the potential of the microbial world in new and unprecedented ways. Some argue that the potential of such microbial revolution is akin to the twentieth century electronic revolution (Curtis, 2006) that have transformed our technological environment. The theoretical and engineering solutions required to realize the proposed SLMC approach are clearly lagging behind the raid advancement in new genetic tools, nevertheless, the concepts presented here are realizable with present methods and we expect that as more aspects of such approach are being tested and shown to offer benefits, new solutions will be developed and scalable systems will become available for research and for many other applications in the fields of food industry, medical, environmental and more.

## Author contributions

All authors listed, have made substantial, direct and intellectual contribution to the work, and approved it for publication.

### Conflict of interest statement

The authors declare that the research was conducted in the absence of any commercial or financial relationships that could be construed as a potential conflict of interest.

## References

[B1] Agency U. E. P. (2002). Nitrification. Technical Report, U.S. Environmental Protection Agency.

[B2] Aggarwal N. HogenEsch H. Guo P. North A. Suckow M. Mittal S. K. (1999). Biodegradable alginate microspheres as a delivery system for naked DNA. Can. J. Veterinary Res. 63, 148–152. 10369574 PMC1189535

[B3] Aida T. Yamaguchi K. (1966). Studies on the utilization of hydrocarbons by yeasts part I. J. Agric. Chem. Soc. Jpn. 40, 119–126. 10.1271/nogeikagaku1924.40.3_119

[B4] Alberts B. Johnson A. Lewis J. Raff M. Roberts K. Walter P. (2008). Molecular Biology of the Cell, 5th Edn. New York, NY: Garland Science.

[B5] Alivisatos A. P. Blaser M. J. Brodie E. L. Chun M. Dangl J. L. Donohue T. J.. (2015). A unified initiative to harness Earth's microbiomes. Science 350, 507–508. 10.1126/science.aac8480 26511287

[B6] Allison S. D. Martiny J. B. H. (2008). Resistance, resilience, and redundancy in microbial communities. Proc. Natl. Acad. Sci. U.S.A. 105(Suppl. 1), 11512–11519. 10.1073/pnas.0801925105 18695234 PMC2556421

[B7] Anderson J. R. Chiu D. T. Jackman R. J. Cherniavskaya O. McDonald J. C. Wu H.. (2000). Fabrication of topologically complex three-dimensional microfluidic systems in PDMS by rapid prototyping. Anal. Chem. 72, 3158–3164. 10.1021/ac9912294 10939381

[B8] Angenent L. T. Wrenn B. A. (2008). Optimizing mixed-culture bioprocessing to convert wastes into bioenergy, in Bioenergy, eds Wall J. Harwood C. Demain A. (Washington, DC: ASM Press), 179–194. 10.1128/9781555815547.ch15

[B9] Arrigo K. R. (2005). Marine microorganisms and global nutrient cycles. Nature 437, 349–355. 10.1038/nature04159 16163345

[B10] Bader J. Mast-Gerlach E. Popović M. K. Bajpai R. Stahl U. (2010). Relevance of microbial coculture fermentations in biotechnology. J. Appl. Microbiol. 109, 371–387. 10.1111/j.1365-2672.2009.04659.x 20070440

[B11] Baeshen M. N. Al-Hejin A. M. Bora R. S. Ahmed M. M. M. Ramadan H. A. I. Saini K. S.. (2015). Production of biopharmaceuticals in *E. coli*: current scenario and future perspectives. J. Microbiol. Biotechnol. 25, 953–962. 10.4014/jmb.1412.12079 25737124

[B12] Baeshen N. A. Baeshen M. N. Sheikh A. Bora R. S. Ahmed M. M. M. Ramadan H. A. I.. (2014). Cell factories for insulin production. Microb. Cell Factor 13:141. 10.1186/s12934-014-0141-0 25270715 PMC4203937

[B13] Baker K. H. Herson D. S. (1978). Interactions between the diatom *Thallasiosira pseudonanna* and an associated pseudomonad in a mariculture system. Appl. Environ. Microbiol. 35, 791–796. 646360 10.1128/aem.35.4.791-796.1978 PMC242924

[B14] Barta D. J. Chullen C. Vega L. Cox M. R. Aitchison L. T. Lange K. E. (2014). Next generation life support project status, in International Conference on Environmental Systems (ICES 2014), (Tuscon, AZ).

[B15] Becker H. Gärtner C. (2008). Polymer microfabrication technologies for microfluidic systems. Anal. Bioanal. Chem. 390, 89–111. 10.1007/s00216-007-1692-2 17989961

[B16] Bernstein H. C. Carlson R. P. (2012). Microbial consortia engineering for cellular factories: *in vitro* to *in silico* systems. Comput. Struct. Biotechnol. J. 3, 1–8. 10.5936/csbj.201210017 24688677 PMC3962199

[B17] Bollmann A. Lewis K. Epstein S. S. (2007). Incubation of environmental samples in a diffusion chamber increases the diversity of recovered isolates. Appl. Environ. Microbiol. 73, 6386–6390. 10.1128/AEM.01309-07 17720826 PMC2075052

[B18] Bonham-Carter J. Shevitz J. (2011). A brief history of perfusion biomanufacturing. Bioprocess Int. 9, 24–30. Available online at: http://www.bioprocessintl.com/upstream-processing/bioreactors/a-brief-history-of-perfusion-biomanufacturing-322322/

[B19] Boonchan S. Britz M. L. Stanley G. A. (2000). Degradation and mineralization of high-molecular-weight polycyclic aromatic hydrocarbons by defined fungal-bacterial cocultures. Appl. Environ. Microbiol. 66, 1007–1019. 10.1128/AEM.66.3.1007-1019.2000 10698765 PMC91936

[B20] Brenner K. You L. Arnold F. H. (2008). Engineering microbial consortia: a new frontier in synthetic biology. Trends Biotechnol. 26, 483–489. 10.1016/j.tibtech.2008.05.004 18675483

[B21] Bronstein J. L. (1994). Our current understanding of mutualism. Q. Rev. Biol. 69, 31–51. 10.1086/418432

[B22] Brune K. D. Bayer T. S. (2012). Engineering microbial consortia to enhance biomining and bioremediation. Front. Microbiol. 3:203. 10.3389/fmicb.2012.00203 22679443 PMC3367458

[B23] Burmolle M. Webb J. S. Rao D. Hansen L. H. Sorensen S. J. Kjelleberg S. (2006). Enhanced biofilm formation and increased resistance to antimicrobial agents and bacterial invasion are caused by synergistic interactions in multispecies biofilms. Appl. Environ. Microbiol. 72, 3916–3923. 10.1128/AEM.03022-05 16751497 PMC1489630

[B24] Butler I. (1994). Removal of dissolved oxygen from water: a comparison of four common techniques. Talanta 41, 211–215. 10.1016/0039-9140(94)80110-X 18965910

[B25] Butler J. E. He Q. Nevin K. P. He Z. Zhou J. Lovley D. R. (2007). Genomic and microarray analysis of aromatics degradation in Geobacter metallireducens and comparison to a Geobacter isolate from a contaminated field site. BMC Genomics 8:180. 10.1186/1471-2164-8-180 17578578 PMC1924859

[B26] Carvallo L. Carrera J. Chamy R. (2002). Nitrifying activity monitoring and kinetic parameters determination in a biofilm airlift reactor by respirometry. Biotechnol. Lett. 24, 2063–2066. 10.1023/A:1021375523879

[B27] Chandran K. Smets B. F. (2005). Optimizing experimental design to estimate ammonia and nitrite oxidation biokinetic parameters from batch respirograms. Water Res. 39, 4969–4978. 10.1016/j.watres.2005.10.001 16300816

[B28] Chang J. Ossoff S. F. Lobe D. C. Dorfman M. H. Dumais C. M. Qualls R. G.. (1985). UV inactivation of pathogenic and indicator microorganisms. Appl. Environ. Microbiol. 49, 1361–1365. 2990336 10.1128/aem.49.6.1361-1365.1985 PMC241729

[B29] Charcosset C. (2006). Membrane processes in biotechnology: an overview. Biotechnol. Adv. 24, 482–492. 10.1016/j.biotechadv.2006.03.002 16687233

[B30] Charles M. (1985). Fermentation scale-up: problems and possibilities. Trends Biotechnol. 3, 134–139. 10.1016/0167-7799(85)90101-5

[B31] Charley R. Hooper D. McLee A. (1980). Nitrification kinetics in activated sludge at various temperatures and dissolved oxygen concentrations. Water Res. 14, 1387–1396. 10.1016/0043-1354(80)90002-0

[B32] Curtis T. (2006). Microbial ecologists: it's time to ‘go large’. Nat. Rev. Microbiol. 4, 488. 10.1038/nrmicro1455 16835959

[B33] Curtis T. P. Sloan W. T. Scannell J. W. (2002). Estimating prokaryotic diversity and its limits. Proc. Natl. Acad. Sci. U.S.A. 99, 10494–10499. 10.1073/pnas.142680199 12097644 PMC124953

[B34] Daims H. Taylor M. W. Wagner M. (2006). Wastewater treatment: a model system for microbial ecology. Trends Biotechnol. 24, 483–489. 10.1016/j.tibtech.2006.09.002 16971007

[B35] Dalsgaard T. Thamdrup B. Canfield D. E. (2005). Anaerobic ammonium oxidation (anammox) in the marine environment. Res. Microbiol. 156, 457–464. 10.1016/j.resmic.2005.01.011 15862442

[B36] Davies J. (2007). Small molecules: the lexicon of biodiversity. J. Biotechnol. 129, 3–5. 10.1016/j.jbiotec.2006.11.023 17208324

[B37] DeAngelis D. L. Grimm V. (2014). Individual-based models in ecology after four decades. F1000Prime Rep. 6:39. 10.12703/P6-39 24991416 PMC4047944

[B38] DeAngelis D. L. Gross L. J. (1992). Individual-Based Models and Approaches in Ecology: Populations, Communities and Ecosystems. New York, NY: Chapman & Hall.

[B39] Degenhardt O. S. Waters B. Rebelo-Cameirao A. Meyer A. Brunner H. Toltl N. P. (2004). Comparison of the effectiveness of various deaeration techniques. Dissolut. Technol. 11, 6–12. 10.14227/DT110104P6

[B40] Devol A. H. (2003). Nitrogen cycle: solution to a marine mystery. Nature 422, 575–576. 10.1038/422575a 12686985

[B41] Dietz D. Sabra W. Zeng A.-p. (2013). Co-cultivation of Lactobacillus zeae and Veillonella cricetifor the production of propionic acid. AMB Exp. 3:29. 10.1186/2191-0855-3-29 23705662 PMC3699425

[B42] Dochain D. Vanrolleghem P. A. (2001). Dynamical Modelling and Estimation in Wastewater Treatment Processes. London: IWA Publishing.

[B43] Doran P. (2012). Bioprocess Engineering Principles. Oxford: Academic Press.

[B44] Drake J. W. Charlesworth B. Charlesworth D. Crow J. F. (1998). Rates of spontaneous mutation. Genetics 148, 1667–1686. 9560386 10.1093/genetics/148.4.1667 PMC1460098

[B45] Duffy D. C. McDonald J. C. Schueller O. J. A. Whitesides G. M. (1998). Rapid prototyping of microfluidic systems in poly(dimethylsiloxane). Anal. Chem. 70, 4974–4984. 10.1021/ac980656z 21644679

[B46] Grady C. P. L. Daigger G. T. Love N. G. Filipe C. D. M. (2011). Biological Wastewater Treatment, 3rd Edn. London: IWA Publishing.

[B47] Eykamp W. (1995). Chapter 1: Microfiltration and ultrafiltration, in Membrane Separations Technology. Principles and Applications, vol. II, eds Noble R. D. Stern S. A. (Amsterdam: Elsevier), 1–43.

[B48] Ferrari B. C. Binnerup S. J. Gillings M. (2005). Microcolony cultivation on a soil substrate membrane system selects for previously uncultured soil bacteria. Appl. Environ. Microbiol. 71, 8714–8720. 10.1128/AEM.71.12.8714-8720.2005 16332866 PMC1317317

[B49] Foster P. L. (2006). Methods for determining spontaneous mutation rates. Methods Enzymol. 409, 195–213. 10.1016/S0076-6879(05)09012-9 16793403 PMC2041832

[B50] Fraser S. J. Endres C. (2013). Quorus bioreactor: a new perfusion-based technology for microbial cultivation, in Advances in Biochemical Engineering/Biotechnology, vol. 123, eds Eibl D. Eibl R. (Heidelberg: Springer-Verlag), 149–177. 10.1007/10_2013_238 23913132

[B51] Frederickson A. (1977). Behavior of mixed culture of microorganisms. Ann. Rev. Microbiol. 31, 63–87. 10.1146/annurev.mi.31.100177.000431 334047

[B52] Frimat J.-P. Becker M. Chiang Y.-Y. Marggraf U. Janasek D. Hengstler J. G.. (2011). A microfluidic array with cellular valving for single cell co-culture. Lab Chip 11, 231–237. 10.1039/C0LC00172D 20978708

[B53] Fu N. Peiris P. Markham J. Bavor J. (2009). A novel co-culture process with *Zymomonas mobilis* and *Pichia stipitis* for efficient ethanol production on glucose/xylose mixtures. Enzyme Microb. Technol. 45, 210–217. 10.1016/j.enzmictec.2009.04.006

[B54] Garcia-Ochoa F. Gomez E. (2009). Bioreactor scale-up and oxygen transfer rate in microbial processes: an overview. Biotechnol. Adv. 27, 153–176. 10.1016/j.biotechadv.2008.10.006 19041387

[B55] Gehin A. Cailliez C. Petitdemange E. Benoit L. (1996). Studies of clostridium cellulolyticum ATCC 35319 under dialysis and co-culture conditions. Lett. Appl. Microbiol. 23, 208–212. 10.1111/j.1472-765X.1996.tb00067.x 8987692

[B56] Gerhardt P. Gallup D. M. (1963). Dialysis flask for concetrated culture of microorganisms. J. Bacteriol. 86, 919–929. 14080802 10.1128/jb.86.5.919-929.1963 PMC278547

[B57] Gernaey K. Vanrolleghem P. Verstraete W. (1998). On-line estimation of Nitrosomonas kinetic parameters in activated sludge samples using titration in-sensor-experiments. Water Res. 32, 71–80. 10.1016/S0043-1354(97)00185-1

[B58] Gitaitis R. D. (1987). Refinement of lyophilization methodology for storage of large numbers of bacterial strains. Plant Dis. 71:615 10.1094/PD-71-0615

[B59] Gòdia F. Albiol J. Montesinos J. Pérez J. Creus N. Cabello F.. (2002). MELISSA: a loop of interconnected bioreactors to develop life support in Space. J. Biotechnol. 99, 319–330. 10.1016/S0168-1656(02)00222-5 12385718

[B60] Grimm V. Railsback S. F. (2005). Individual-Based Modeling and Ecology. Princeton, NJ: Princeton University Press.

[B61] Großkopf T. Soyer O. S. (2014). Synthetic microbial communities. Curr. Opin. Microbiol. 18, 72–77. 10.1016/j.mib.2014.02.002 24632350 PMC4005913

[B62] Guedon E. Desvaux M. S. P. Petitdemange H. (1999). Growth inhibition of Clostridium cellulolyticum by an inefficiently regulated carbon flow. Microbiology 45, 1831–1838. 10.1099/13500872-145-8-1831 10463149

[B63] Haferburg G. Kothe E. (2010). Metallomics: lessons for metalliferous soil remediation. Appl. Microbiol. Biotechnol. 87, 1271–1280. 10.1007/s00253-010-2695-z 20532755

[B64] Hatzenpichler R. (2012). Diversity, physiology, and niche differentiation of ammonia-oxidizing archaea. Appl. Environ. Microbiol. 78, 7501–7510. 10.1128/AEM.01960-12 22923400 PMC3485721

[B65] Hays S. G. Patrick W. G. Ziesack M. Oxman N. Silver P. A. (2015). Better together: engineering and application of microbial symbioses. Curr. Opin. Biotechnol. 36, 40–49. 10.1016/j.copbio.2015.08.008 26319893

[B66] He Y. Xie K. Xu P. Huang X. Gu W. Zhang F.. (2013). Evolution of microbial community diversity and enzymatic activity during composting. Res. Microbiol. 164, 189–198. 10.1016/j.resmic.2012.11.001 23178379

[B67] Hellinga C. van Loosdrecht M. Heijnen J. (1999). Model based design of a novel process for nitrogen removal from concentrated flows. Math. Comput. Model. Dyn. Syst. 5, 351–371. 10.1076/mcmd.5.4.351.3678

[B68] Hendrickx L. De Wever H. Hermans V. Mastroleo F. Morin N. Wilmotte A.. (2006). Microbial ecology of the closed artificial ecosystem MELiSSA (Micro-ecological life support system alternative): reinventing and compartmentalizing the Earth's food and oxygen regeneration system for long-haul space exploration missions. Res. Microbiol. 157, 77–86. 10.1016/j.resmic.2005.06.014 16431089

[B69] Henry C. S. Broadbelt L. J. Hatzimanikatis V. (2007). Thermodynamics-based metabolic flux analysis. Biophys. J. 92, 1792–1805. 10.1529/biophysj.106.093138 17172310 PMC1796839

[B70] Henze M. Harremoës P. Cour Jansen J. Arvin E. (1997). Wastewater Treatment. Berlin; Heidelberg: Springer.

[B71] Hijnen W. Beerendonk E. Medema G. (2006). Inactivation credit of UV radiation for viruses, bacteria and protozoan (oo)cysts in water: a review. Water Res. 40, 3–22. 10.1016/j.watres.2005.10.030 16386286

[B72] Hong S. Pan Q. Lee L. P. (2012). Single-cell level co-culture platform for intercellular communication. Integr. Biol. 4:374. 10.1039/c2ib00166g 22434268

[B73] Hong S. H. Hegde M. Kim J. Wang X. Jayaraman A. Wood T. K. (2012). Synthetic quorum-sensing circuit to control consortial biofilm formation and dispersal in a microfluidic device. Nat. Commun. 3:613. 10.1038/ncomms1616 22215088 PMC3272573

[B74] Horst W. D. Rokem J. S. Berovic M. (2009). Biotechnology. Oxford: Eolss Publishers.

[B75] Hunik J. H. Bos C. G. van den Hoogen M. P. De Gooijer C. D. Tramper J. (1994). Co-immobilizedNitrosomonas europaea andNitrobacter agilis cells: validation of a dynamic model for simultaneous substrate conversion and growth in K-carrageenan gel beads. Biotechnol. Bioeng. 43, 1153–1163. 10.1002/bit.260431121 18615529

[B76] Ihssen J. Kowarik M. Dilettoso S. Tanner C. Wacker M. Thöny-Meyer L. (2010). Production of glycoprotein vaccines in *Escherichia coli*. Microb. Cell Factor. 9:61. 10.1186/1475-2859-9-61 20701771 PMC2927510

[B77] Jagmann N. Philipp B. (2014). Reprint of Design of synthetic microbial communities for biotechnological production processes. J. Biotechnol. 192, 293–301. 10.1016/j.jbiotec.2014.11.005 25444870

[B78] Jeong H.-H. Jin S. H. Lee B. J. Kim T. Lee C.-S. (2015). Microfluidic static droplet array for analyzing microbial communication on a population gradient. Lab Chip 15, 889–899. 10.1039/C4LC01097C 25494004

[B79] Jetten M. S. Wagner M. Fuerst J. van Loosdrecht M. Kuenen G. Strous M. (2001). Microbiology and application of the anaerobic ammonium oxidation (‘anammox’) process. Curr. Opin. Biotechnol. 12, 283–288. 10.1016/S0958-1669(00)00211-1 11404106

[B80] Johns N. I. Blazejewski T. Gomes A. L. Wang H. H. (2016). Principles for designing synthetic microbial communities. Curr. Opin. Microbiol. 31, 146–153. 10.1016/j.mib.2016.03.010 27084981 PMC4899134

[B81] Jones S. B. Or D. Heinse R. Tuller M. (2012). Beyond earth: designing root zone environments for reduced gravity conditions. Vadose Zone J. 11, 11–21. 10.2136/vzj2011.0081

[B82] Kalscheuer R. (2006). Microdiesel: *Escherichia coli* engineered for fuel production. Microbiology 152, 2529–2536. 10.1099/mic.0.29028-0 16946248

[B83] Kamionka M. (2011). Engineering of therapeutic proteins production in *Escherichia coli*. Curr. Pharm. Biotechnol. 12, 268–274. 10.2174/138920111794295693 21050165 PMC3179032

[B84] Kartal B. Kuenen J. G. van Loosdrecht M. C. M. (2010). Sewage treatment with anammox. Science 328, 702–703. 10.1126/science.1185941 20448175

[B85] Keen G. A. Prosser J. I. (1987). Steady state and transient growth of autotrophic nitrifying bacteria. Arch. Microbiol. 147, 73–79. 10.1007/BF00492908

[B86] Kim H. J. Boedicker J. Q. Choi J. W. Ismagilov R. F. (2008). Defined spatial structure stabilizes a synthetic multispecies bacterial community. Proc. Natl. Acad. Sci. U.S.A. 105, 18188–18193. 10.1073/pnas.0807935105 19011107 PMC2587551

[B87] Kim J.-D. Lee C.-G. (2007). Microbial degradation of polycyclic aromatic hydrocarbons in soil by bacterium-fungus co-cultures. Biotechnol. Bioproc. Eng. 12, 410–416. 10.1007/BF02931064

[B88] Kleerebezem R. van Loosdrecht M. C. (2007). Mixed culture biotechnology for bioenergy production. Curr. Opin. Biotechnol. 18, 207–212. 10.1016/j.copbio.2007.05.001 17509864

[B89] Klein J. Stock J. Vorlop K. D. (1983). Pore size and properties of spherical Ca-alginate biocatalysts. Eur. J. Appl. Microb. Biotechnol. 18, 86–91. 10.1007/BF00500829

[B90] Konopka A. (2009). What is microbial community ecology? ISME J. 3, 1223–1230. 10.1038/ismej.2009.88 19657372

[B91] Konopka A. Lindemann S. Fredrickson J. (2015). Dynamics in microbial communities: unraveling mechanisms to identify principles. ISME J. 9, 1488–1495. 10.1038/ismej.2014.251 25526370 PMC4478703

[B92] Koops H. (2001). Distribution and ecophysiology of the nitrifying bacteria emphasizing cultured species. FEMS Microbiol. Ecol. 37, 1–9. 10.1111/j.1574-6941.2001.tb00847.x

[B93] Kreft J.-U. Booth G. Wimpenny J. W. T. (1998). BacSim, a simulator for individual-based modelling of bacterial colony growth. Microbiology 144, 3275–3287. 10.1099/00221287-144-12-3275 9884219

[B94] Kreft J. U. Picioreanu C. Wimpenny J. W. van Loosdrecht M. C. (2001). Individual-based modelling of biofilms. Microbiology (Reading, England) 147(Pt 11), 2897–2912. 10.1099/00221287-147-11-2897 11700341

[B95] Krömer J. O. Nielsen L. Blank L. M. (2014). Metabolic Flux Analysis in Eukaryotes. Methods in Molecular Biology. New York, NY: Springer.

[B96] Kuenen J. G. (2008). Anammox bacteria: from discovery to application. Nat. Rev. Microbiol. 6, 320–326. 10.1038/nrmicro1857 18340342

[B97] Kuo A. C. M. (1999). Poly (dimethylsiloxane), in Polymer Data Handbook, ed Mark J. E. (New York, NY: Oxford University Press), 411–435.

[B98] Kuypers M. M. M. Sliekers A. O. Lavik G. Schmid M. Jørgensen B. B. Kuenen J. G.. (2003). Anaerobic ammonium oxidation by anammox bacteria in the Black Sea. Nature 422, 608–611. 10.1038/nature01472 12686999

[B99] Lamberti A. Marasso S. L. Cocuzza M. (2014). PDMS membranes with tunable gas permeability for microfluidic applications. RSC Adv. 4, 61415–61419. 10.1039/C4RA12934B

[B100] Langer E. S. (2011). Trends in perfusion bioreactors: the next revolution in bioprocessing? Bioproc. Int. 9, 18–22. Available online at: http://www.bioprocessintl.com/upstream-processing/bioreactors/trends-in-perfusion-bioreactors-323459/

[B101] Langer E. S. Rader R. A. (2014). Continuous bioprocessing and perfusion: wider adoption coming as bioprocessing matures. BioProcess. J. 13, 43–49. 10.12665/J131.Langer

[B102] Lardon L. A. Merkey B. V. Martins S. Dötsch A. Picioreanu C. Kreft J.-U.. (2011). iDynoMiCS: next-generation individual-based modelling of biofilms. Environ. Microbiol. 13, 2416–2434. 10.1111/j.1462-2920.2011.02414.x 21410622

[B103] Lasseur C. Brunet J. de Weever H. Dixon M. Dussap G. Godia F. (2010). Melissa: the European project of closed life support system. Gravitat. Space Biol. 23, 3–12. Available online at: http://gravitationalandspacebiology.org/index.php/journal/article/view/487

[B104] Lee K. Y. Heo T. R. (2000). Survival of Bifidobacterium longum immobilized in calcium alginate beads in simulated gastric juices and bile salt solution. Appl. Environ. Microbiol. 66, 869–873. 10.1128/AEM.66.2.869-873.2000 10653768 PMC91913

[B105] Lee K. Y. Mooney D. J. (2012). Alginate: properties and biomedical applications. Prog. Polym. Sci. 37, 106–126. 10.1016/j.progpolymsci.2011.06.003 22125349 PMC3223967

[B106] Leung K. Zahn H. Leaver T. Konwar K. M. Hanson N. W. Page A. P.. (2012). A programmable droplet-based microfluidic device applied to multiparameter analysis of single microbes and microbial communities. Proc. Natl. Acad. Sci. U.S.A. 109, 7665–7670. 10.1073/pnas.1106752109 22547789 PMC3356603

[B107] Li L. Shi M. Song Y. Bao L. Yang W. Zhang X. (2009). A single-use, scalable perfusion bioreactor system. Bioproc. Int. 7, 46–54. Available online at: http://www.bioprocessintl.com/upstream-processing/upstream-single-use-technologies/a-single-use-scalable-perfusion-bioreactor-system-182113/

[B108] Lin H. Wang Q. Shen Q. Zhan J. Zhao Y. (2013). Genetic engineering of microorganisms for biodiesel production. Bioengineered 4, 292–304. 10.4161/bioe.23114 23222170 PMC3813529

[B109] Lindemann S. R. Bernstein H. C. Song H.-S. Fredrickson J. K. Fields M. W. Shou W.. (2016). Engineering microbial consortia for controllable outputs. ISME J. 10, 2077–2084. 10.1038/ismej.2016.26 26967105 PMC4989317

[B110] Locey K. J. Lennon J. T. (2016). Scaling laws predict global microbial diversity. Proc. Natl. Acad. Sci. U.S.A. 113, 5970–5975. 10.1073/pnas.1521291113 27140646 PMC4889364

[B111] Lovley D. R. (2003). Cleaning up with genomics: applying molecular biology to bioremediation. Nat. Rev. Microbiol. 1, 35–44. 10.1038/nrmicro731 15040178

[B112] Lovley D. R. Giovannoni S. J. White D. C. Champine J. E. Phillips E. J. P. Gorby Y. A.. (1993). Geobacter metallireducens gen. nov. sp. nov., a microorganism capable of coupling the complete oxidation of organic compounds to the reduction of iron and other metals. Arch. Microbiol. 159, 336–344. 10.1007/BF00290916 8387263

[B113] Luo X. Tsao C.-Y. Wu H.-C. Quan D. N. Payne G. F. Rubloff G. W.. (2015). Distal modulation of bacterial cell–cell signalling in a synthetic ecosystem using partitioned microfluidics. Lab Chip 15, 1842–1851. 10.1039/C5LC00107B 25690330

[B114] Luthy R. G. Aiken G. R. Brusseau M. L. Cunningham S. D. Gschwend P. M. Pignatello J. J. (1997). Sequestration of hydrophobic organic contaminants by geosorbents. Environ. Sci. Technol. 31, 3341–3347. 10.1021/es970512m

[B115] Lynd L. R. Weimer P. J. van Zyl W. H. Pretorius I. S. (2002). Microbial cellulose utilization: fundamentals and biotechnology. Microbiol. Mol. Biol. Rev. 66, 506–577. 10.1128/MMBR.66.3.506-577.2002 12209002 PMC120791

[B116] Maggi F. Pallud C. (2010a). Martian base agriculture: the effect of low gravity on water flow, nutrient cycles, and microbial biomass dynamics. Adv. Space Res. 46, 1257–1265. 10.1016/j.asr.2010.07.012

[B117] Maggi F. Pallud C. (2010b). Space agriculture in micro- and hypo-gravity: a comparative study of soil hydraulics and biogeochemistry in a cropping unit on Earth, Mars, the Moon and the space station. Planet. Space Sci. 58, 1996–2007. 10.1016/j.pss.2010.09.025

[B118] Maki M. Leung K. T. Qin W. (2009). The prospects of cellulase-producing bacteria for the bioconversion of lignocellulosic biomass. Int. J. Biol. Sci. 5, 500–516. 10.7150/ijbs.5.500 19680472 PMC2726447

[B119] Manjarrez E. S. Albasi C. Riba J.-P. (2000). A two-reservoir, hollow-fiber bioreactor for the study of mixed-population dynamics: design aspects and validation of the approach. Biotechnol. Bioeng. 69, 401–408. 10.1002/1097-0290(20000820)69:43.0.CO;2-3 10862678

[B120] Matson S. L. (1995). Chapter 8: Membrane bioseparations, in Membrane Science and Technology, Vol. 2, Noble R. D. Stern S. A. (Amsterdam: Elsevier), 353–413. 10.1016/s0927-5193(06)80010-0

[B121] Mazur P. (1984). Freezing of living cells: mechanisms and implications. Am. J. Physiol. 247(3 Pt 1), C125–C142. 6383068 10.1152/ajpcell.1984.247.3.C125

[B122] McDonald (1993). In Situ Bioremediation. Washington, DC: National Academies Press.

[B123] McDonald J. C. Duffy D. C. Anderson J. R. Chiu D. T. Wu H. Schueller O. J. A.. (2000). Fabrication of microfluidic systems in poly(dimethylsiloxane). Electrophoresis 21, 27–40. 10.1002/(SICI)1522-2683(20000101)21:1<27::AID-ELPS27>3.0.CO;2-C 10634468

[B124] McDonald J. C. Whitesides G. M. (2002). Poly(dimethylsiloxane) as a material for fabricating microfluidic devices. Accounts Chem. Res. 35, 491–499. 10.1021/ar010110q 12118988

[B125] Menezes A. A. Cumbers J. Hogan J. A. Arkin A. P. (2014). Towards synthetic biological approaches to resource utilization on space missions. J. R. Soc. Interf. 12:20140715. 10.1098/rsif.2014.0715 25376875 PMC4277073

[B126] Metcalf & Eddy Inc. Tchobanoglous G. Burton F. L. Stensel H. D. (2003). Wastewater Engineering: Treatment and Reuse, 4th Edn. Boston, MA: McGraw-Hill.

[B127] Miralles V. Huerre A. Malloggi F. Jullien M.-C. (2013). A review of heating and temperature control in microfluidic systems: techniques and applications. Diagnostics 3, 33–67. 10.3390/diagnostics3010033 26835667 PMC4665581

[B128] Mohan R. Sanpitakseree C. Desai A. V. Sevgen S. E. Schroeder C. M. Kenis P. J. A. (2015). A microfluidic approach to study the effect of bacterial interactions on antimicrobial susceptibility in polymicrobial cultures. RSC Adv. 5, 35211–35223. 10.1039/C5RA04092B

[B129] Morgan C. Herman N. White P. Vesey G. (2006). Preservation of micro-organisms by drying; a review. J. Microbiol. Methods 66, 183–193. 10.1016/j.mimet.2006.02.017 16632005

[B130] Nichols D. Cahoon N. Trakhtenberg E. M. Pham L. Mehta A. Belanger A.. (2010). Use of ichip for high-throughput *in situ* cultivation of “Uncultivable” microbial species. Appl. Environ. Microbiol. 76, 2445–2450. 10.1128/AEM.01754-09 20173072 PMC2849220

[B131] Nowka B. Daims H. Spieck E. (2015). Comparison of oxidation kinetics of nitrite-oxidizing bacteria: nitrite availability as a key factor in niche differentiation. Appl. Environ. Microbiol. 81, 745–753. 10.1128/AEM.02734-14 25398863 PMC4277589

[B132] Ohno M. Okano I. Watsuji T.-O. Kakinuma T. Ueda K. Beppu T. (1999). Establishing the independent culture of a strictly symbiotic bacterium symbiobacterium thermophilum from its supporting bacillus strain. Biosci. Biotechnol. Biochem. 63, 1083–1090. 10.1271/bbb.63.1083 10427695

[B133] Orth J. D. Conrad T. M. Na J. Lerman J. A. Nam H. Feist A. M.. (2011). A comprehensive genome-scale reconstruction of *Escherichia coli* metabolism–2011. Mol. Syst. Biol. 7, 535. 10.1038/msb.2011.65 21988831 PMC3261703

[B134] Orth J. D. Thiele I. Palsson B. Ø. (2010). What is flux balance analysis? Nat. Biotechnol. 28, 245–248. 10.1038/nbt.1614 20212490 PMC3108565

[B135] Painter H. (1970). A review of literature on inorganic nitrogen metabolism in microorganisms. Water Res. 4, 393–450. 10.1016/0043-1354(70)90051-5

[B136] Palomares L. A. Ramirez O. T. (2000). Bioreactor scale-up, in The Encyclopedia of Cell Technology, Vol 1, ed Spier R. E. (John Wiley and Sons) 183–201.

[B137] Park S. Bae W. (2009). Modeling kinetics of ammonium oxidation and nitrite oxidation under simultaneous inhibition by free ammonia and free nitrous acid. Proc. Biochem. 44, 631–640. 10.1016/j.procbio.2009.02.002

[B138] Pohlscheidt M. Charaniya S. Bork C. Jenzsch M. Noetzel T. L. Luebbert A. (2013). Bioprocess and fermentation monitoring, in Encyclopedia of Industrial Biotechnology, ed Flickinger M. C. (Hoboken, NJ: John Wiley & Sons, Inc.), 1469–1492.

[B139] Poltak R. F. (2005). Sequencing Batch Reactor Design and Operational Considerations Manual. New England Interstate Water Pollution Control Commission: Massachusetts, September, 27.

[B140] Pörtner R. Märkl H. (1998). Dialysis cultures. Appl. Microbiol. Biotechnol. 50, 403–414. 10.1007/s002530051312 9830090

[B141] Prosser J. I. (2005). Nitrogen in soils: nitrification. Encyclopedia Soils Environ. 1, 31–39. 10.1016/B0-12-348530-4/00512-9

[B142] Reisman H. B. (1993). Problems in scale-up of biotechnology production processes. Crit. Rev. Biotechnol. 13, 195–253. 10.3109/07388559309041319 8221898

[B143] Ren K. Zhou J. Wu H. (2013). Materials for microfluidic chip fabrication. Accounts Chem. Res. 46, 2396–2406. 10.1021/ar300314s 24245999

[B144] Renda B. A. Hammerling M. J. Barrick J. E. (2014). Engineering reduced evolutionary potential for synthetic biology. Mol. Biosyst. 10, 1668. 10.1039/c3mb70606k 24556867 PMC4043932

[B145] Robert I. L. Arthur W. B. (1979). Sequencing batch biological reactors: an overview. J. Water Poll. Cont. Federation 51, 235–243.

[B146] Robles-González I. V. Fava F. Poggi-Varaldo H. M. (2008). A review on slurry bioreactors for bioremediation of soils and sediments. Microb. Cell Factor. 7:5. 10.1186/1475-2859-7-5 18312630 PMC2292675

[B147] Rosche W. A. Foster P. L. (2000). Determining mutation rates in bacterial populations. Methods 20, 4–17. 10.1006/meth.1999.0901 10610800 PMC2932672

[B148] Rosenberg E. DeLong E. F. Thompson F. Lory S. Stackebrandt E. (2013). The Prokaryotes. Berlin; Heidelberg: Springer.

[B149] Rowan N. J. MacGregor S. J. Anderson J. G. Fouracre R. A. McIlvaney L. Farish O. (1999). Pulsed-light inactivation of food-related microorganisms. Appl. Environ. Microbiol. 65, 1312–1315. 10049899 10.1128/aem.65.3.1312-1315.1999 PMC91180

[B150] Sabra W. Dietz D. Tjahjasari D. Zeng A. P. (2010). Biosystems analysis and engineering of microbial consortia for industrial biotechnology. Eng. Life Sci. 10, 407–421. 10.1002/elsc.201000111

[B151] Salgado E. Albasi C. Riba J. P. (1998). Mise en oeuvre d'un réacteur à membranes fibres creuses pour l'étude de la dynamique de populations mixtes. Microbiol. Aliments Nutr. 16, 113–120.

[B152] Schellenberger J. Que R. Fleming R. M. T. Thiele I. Orth J. D. Feist A. M.. (2011). Quantitative prediction of cellular metabolism with constraint-based models: the COBRA Toolbox v2.0. Nat. Protocols 6, 1290–1307. 10.1038/nprot.2011.308 21886097 PMC3319681

[B153] Scherer P. Kluge M. Klein J. Sahm H. (1981). Immobilization of the methanogenic bacterium *Methanosarcina barkeri*. Biotechnol. Bioeng. 23, 1057–1065. 10.1002/bit.260230513

[B154] Shevitz J. Bonham-Carter J. Sinclair A. Lim J. (2011). An economic comparison of three cell culture techniques. Biopharm Int. 24, 1–6. Available online at: http://www.biopharminternational.com/economic-comparison-three-cell-culture-techniques

[B155] Shieh W. K. LaMotta E. J. (1979). The intrinsic kinetics of nitrification in a continuous flow suspended growth reactor. Water Res. 13, 1273–1279. 10.1016/0043-1354(79)90171-4

[B156] Skaalure S. C. (2008). Characterization of sterilization techniques on a microfluidic oxygen delivery device. J. Undergraduate Res. 2, 1–4. Available online at: http://firstmonday.org/ojs/index.php/JUR/article/view/7462/5946

[B157] Soh K. C. Hatzimanikatis V. (2014). Constraining the flux space using thermodynamics and integration of metabolomics data, in Methods in Molecular Biology (Clifton, N.J.), vol. 1191 (New York, NY: Springer), 49–63. 10.1007/978-1-4939-1170-7_3 25178783

[B158] Soliman E. A. El-Moghazy A. Y. El-Din M. S. M. Massoud M. A. (2013). Microencapsulation of essential oils within alginate: formulation and *in vitro* evaluation of antifungal activity. J. Encapsul. Adsorp. Sci. 3, 48–55. 10.4236/jeas.2013.31006

[B159] Stanbury P. Whitaker A. Hall S. (2017). Principles of Fermentation Technology, 3rd Edn. Oxford: Elsevier.

[B160] Stewart E. J. (2012). Growing unculturable bacteria. J. Bacteriol. 194, 4151–4160. 10.1128/JB.00345-12 22661685 PMC3416243

[B161] Strous M. Van Gerven E. Kuenen J. G. Jetten M. (1997). Effects of aerobic and microaerobic conditions on anaerobic ammonium-oxidizing (anammox) sludge. Appl. Environ. Microbiol. 63, 2446–2448. 16535633 10.1128/aem.63.6.2446-2448.1997 PMC1389188

[B162] Sun Y. Cheng J. (2002). Hydrolysis of lignocellulosic materials for ethanol production: a review. Bioresour. Technol. 83, 1–11. 10.1016/S0960-8524(01)00212-7 12058826

[B163] Tannenbaum M. Kornfeld J. M. (1975). Multiple Diffusion Chamber. US Patent 3893891 New Brunswick, NJ: Google Patents.

[B164] Tiedje J. M. Colwell R. K. Grossman Y. L. Hodson R. E. Lenski R. E. Mack R. N. (1989). The planned introduction of genetically engineered organisms: ecological considerations and recommendations. Ecology 70, 298–315. 10.2307/1937535

[B165] Turley C. Lochte K. (1985). Direct measurement of bacterial productivity in stratified waters close to a front in the Irish Sea. Mar. Ecol. Prog. Ser. 23, 209–219. 10.3354/meps023209

[B166] Ueda K. Saka H. Ishikawa Y. Kato T. Takeshita Y. Shiratori H.. (2002). Development of a membrane dialysis bioreactor and its application to a large-scale culture of a symbiotic bacterium, *Symbiobacterium thermophilum*. Appl. Microbiol. Biotechnol. 60, 300–305. 10.1007/s00253-002-1117-2 12436311

[B167] Ueng S. W. Lee M. S. Lin S. Chan E.-C. Liu S.-J. (2007). Development of a biodegradable alginate carrier system for antibiotics and bone cells. J. Orthop. Res. 25, 62–72. 10.1002/jor.20286 17019681

[B168] Unger M. A. (2000). Monolithic microfabricated valves and pumps by multilayer soft lithography. Science 288, 113–116. 10.1126/science.288.5463.113 10753110

[B169] Vadivelu V. M. Yuan Z. Fux C. Keller J. (2006). Stoichiometric and kinetic characterisation of Nitrobacter in mixed culture by decoupling the growth and energy generation processes. Biotechnol. Bioeng. 94, 1176–1188. 10.1002/bit.20956 16673416

[B170] van Dongen U. Jetten M. S. M. van Loosdrecht M. C. M. (2001). The SHARON-Anammox process for treatment of ammonium rich wastewater. Water Sci. Technol. 44, 153–160. Available online at: http://wst.iwaponline.com/content/44/1/153 11496667

[B171] van Haandel A. van der Lubbe J. (2012). Handbook of Biological Wastewater Treatment: Design and Optimisation of Activated Sludge Systems. London: IWA Publishing.

[B172] van Reis R. Zydney A. (2001). Membrane separations in biotechnology. Curr. Opin. Biotechnol. 12, 208–211. 10.1016/S0958-1669(00)00201-9 11287239

[B173] van Reis R. Zydney A. (2007). Bioprocess membrane technology. J. Memb. Sci. 297, 16–50. 10.1016/j.memsci.2007.02.045

[B174] Vidali M. (2001). Bioremediation. An overview. Pure Appl. Chem. 73, 1163–1172. 10.1351/pac200173071163

[B175] Villadsen J. Nielsen J. Lidén G. (2011). Bioreaction Engineering Principles, 3rd Edn., Vol. 25. Boston, MA: Springer US.

[B176] Wacker M. Linton D. Hitchen P. G. Nita-Lazar M. Haslam S. M. North S. J.. (2002). N-linked glycosylation in campylobacter jejuni and its functional transfer into *E. coli*. Science 298, 1790–1793. 10.1126/science.298.5599.1790 12459590

[B177] Wang J. (2006). Analytical Electrochemistry, vol. 7 Hoboken, NJ: John Wiley & Sons, Inc.

[B178] Wang Z. Wu X. Peng J. Hu Y. Fang B. Huang S. (2014). Artificially constructed quorum-sensing circuits are used for subtle control of bacterial population density. PLoS ONE 9:e104578. 10.1371/journal.pone.0104578 25119347 PMC4132116

[B179] Ward B. (1996). Nitrification and denitrification: probing the nitrogen cycle in aquatic environments. Microb. Ecol. 32, 247–261. 10.1007/BF00183061 8849421

[B180] Ward B. B. (2008). Nitrification, in Encyclopedia of Ecology, eds Jorgensen S. E. Fath B. (Amsterdam), 2511–2518. 10.1016/B978-008045405-4.09004-2

[B181] Whitesides G. M. Ostuni E. Takayama S. Jiang X. Ingber D. E. (2001). Soft lithography in biology and biochemistry. Ann. Rev. Biomed. Eng. 3, 335–373. 10.1146/annurev.bioeng.3.1.335 11447067

[B182] Whitford W. G. Cadwell J. J. S. (2009). Interest in hollow-fiber perfusion bioreactors is growing. Bioproc. Int. 7, 54–64.

[B183] Wiesler F. Sodaro R. (1996). Deaeration - degasification of water using novel membrane technology. Ultrapure Water 13, 53–56.

[B184] Wiesmann U. (1994). Biological nitrogen removal from wastewater, in Advances in Biochemical Engineering/Biotechnology, vol. 51, ed Fiechter A. (Weinheim: Wiley-VCH Verlag GmbH & Co. KGaA), 113–154. 10.1007/BFb0008736 8165950

[B185] Wijffels R. H. (2015). Biosafety and the Environmental Uses of Micro-Organisms. Harmonisation of Regulatory Oversight in Biotechnology. Paris: OECD Publishing.

[B186] Wijffels R. H. de Gooijer C. D. Kortekaas S. Tramper J. (1991). Growth and substrate consumption ofNitrobacter agilis cells immobilized in carrageenan: part 2. Model evaluation. Biotechnol. Bioeng. 38, 232–240. 10.1002/bit.260380304 18600756

[B187] Yavuz C. Oliaei S. N. B. Cetin B. Yesil-Celiktas O. (2016). Sterilization of PMMA microfluidic chips by various techniques and investigation of material characteristics. J. Supercrit. Fluids 107, 114–121. 10.1016/j.supflu.2015.08.019

[B188] You L. Cox R. S. Weiss R. Arnold F. H. (2004). Programmed population control by cell–cell communication and regulated killing. Nature 428, 868–871. 10.1038/nature02491 15064770

[B189] Zaldivar J. Nielsen J. Olsson L. (2001). Fuel ethanol production from lignocellulose: a challenge for metabolic engineering and process integration. Appl. Microbiol. Biotechnol. 56, 17–34. 10.1007/s002530100624 11499926

[B190] Zehr J. P. Kudela R. M. (2011). Nitrogen cycle of the open ocean: from genes to ecosystems. Ann. Rev. Mar. Sci. 3, 197–225. 10.1146/annurev-marine-120709-142819 21329204

[B191] Zhang H. Wang X. (2016). Modular co-culture engineering, a new approach for metabolic engineering. Metab. Eng. 37, 114–121. 10.1016/j.ymben.2016.05.007 27242132

[B192] Zhang R. Huang J. Xie F. Wang B. Chu M. Wang Y.. (2014). Microfluidic sterilization. Biomicrofluidics 8:034119. 10.1063/1.4882776 25379079 PMC4162453

[B193] Zuroff T. R. Curtis W. R. (2012). Developing symbiotic consortia for lignocellulosic biofuel production. Appl. Microbiol. Biotechnol. 93, 1423–1435. 10.1007/s00253-011-3762-9 22278256

