# Supplementary material for: Synthetic Microbial Ecology: Engineering Habitats for Modular Consortia
Source: Front Microbiol. 2017 Jun 16;8:1125. doi: 10.3389/fmicb.2017.01125 (PMC5472676; doi:10.3389/fmicb.2017.01125)
Supplement: Supplementary file 1 [file Presentation1.PDF]

Supplementary Material

Synthetic microbial ecology: engineering habitats for modular consortia

Sami Ben Said[1] and Dani Or[1]

[1]Department of Environmental Systems Science, ETH Zürich, Zürich, Switzerland

Appendix

This model aimed at comparing the ability of a consortium of two nitrifying bacteria to convert ammonia to nitrate using two different bioreactor layouts. The two microbial species *Nitrosomonas sp.* and *Nitrobacter sp.* are involved in the nitrification process, part of the nitrogen cycle (see figure "applications" in main text) and are important actors for nitrogen removal in wastewater treatment [1–5]. *Nitromonas* is responsible for the nitritation process during which NH_3 is converted to NO_2^-. In a second step, *Nitrobacter* oxidizes NO_2^- to NO_3^- in a process called nitratation (Table 1 and Fig. 1).
In the co-culture simulation, both species were cultured in the same bioreactor, whereas the sequential approach contained a monoculture of *Nitrosomonas* in the first bioreactor which oxidized NH_3 to NO_2^-, the content of which was fed into the second bioreactor containing only *Nitrobacter* for the final oxidation of NO_2^- to NO_3^- (see Reactions, Fig.1). The objective was to compare both layouts for their ability to convert NH_3 to NO_3^-.
In a first step we simulated both scenarios (co-culture and sequential) under the same environmental conditions (i.e. same T° and pH). The conditions in the sequential bioreactors were then improved by tuning each bioreactor separately to provide the cells with better conditions than in co-culture (Fig.1). In each case the cells were confined into their respective bioreactors (no biomass entered nor exited the bioreactors). It was assumed that ammonia (NH_3), nitrite (NO_2^-) and oxygen were the only limiting substrates. The fluxes in and out of the bioreactors were identical and constant. Temperature and pH were kept constant. Although the nitritation and nitratation processes are multistep processes [6, 7], we used simplified chemical reactions corresponding to the sum of their respective partial reactions (Table 1).
The dynamic processes included in this model are biomass growth, cellular maintenance and biomass decay. Growth was limited and inhibited by the nitrogen substrates as well as limited by the availability of oxygen. A symmetrical inhibition term resulting in the inhibition of the growth of one member by the substrate of the other member was also considered. A final dependence of microbial growth was added by including a pH dependence term. The following ODE systems were solved with MATLAB R2016a.
For additional information please contact Sami Ben Said (`sbensaid@usys.ethz.ch`).

Reaction	Description	Refs
$NH_3 + 1.5\,O_2 \longrightarrow {NO_2}^- + H^+ + H_2O$	Ammonia oxidation by *Nitrosomonas*	[3, 8–12]
${NO_2}^- + 0.5\,O_2 \longrightarrow {NO_3}^-$	Nitrite oxidation by *Nitrobacter*	[3, 8–12]
${NH_4}^+ \rightleftarrows NH_3 + H^+$	Dissociation equilibrium between ammonium and ammonia	[13]
$HNO_2 \rightleftarrows {NO_2}^- + H^+$	Dissociation equilibrium between nitrite and nitrous acid	[13]

Table 1: **Reactions**

Enzymatic oxidation of ammonia (NH_3) by *Nitrosomonas* and nitrite (NO_2^-) by *Nitrobacter* as well as their dissociation equilibria.

Equation	Description	Refs
$\mathcal{S}_\mathrm{S}^\mathrm{X} = \frac{[\mathrm{S}]}{[\mathrm{S}] + \mathrm{K_S} + \frac{[\mathrm{S}]^2}{\mathrm{K}_{\mathrm{I_S}}^\mathrm{X}}}$ (1)	Substrate-limited/inhibited growth	[1, 14–20]
$\mathcal{I}_\mathrm{i}^\mathrm{X} = \frac{\mathrm{K}_{\mathrm{I_i}}}{\mathrm{K}_{\mathrm{I_i}} + [\mathrm{i}]}$ (2)	Additional inhibitions (other than substrate inhibition)	[1, 14, 16, 17, 20, 21]
$\mathcal{L}_\mathrm{S}^\mathrm{X} = \frac{[\mathrm{S}]}{\mathrm{K}_\mathrm{S}^\mathrm{X} + [\mathrm{S}]}$ (3)	Additional limiting substrates	[22–25]
$\mathcal{L}_\mathrm{pH} = \frac{\mathrm{K_{pH}}}{\mathrm{K_{pH}} + 10^{\lvert \mathrm{pH_{opt}} - \mathrm{pH} \rvert} - 1}$ (4)	pH dependent growth	[2, 4, 17, 24–26]
$[\mathrm{NH_3}] = \frac{\mathrm{Ka}_{\mathrm{NH_4^+}}}{\mathrm{Ka}_{\mathrm{NH_4^+}} + [\mathrm{H^+}]} \cdot [\mathrm{TAN}]$ (5)	Ammonia concentration as a function of pH, dissociation constant and total ammonia nitrogen (TAN)	[17]
$[\mathrm{HNO_2}] = \frac{[\mathrm{H^+}]}{\mathrm{Ka_{HNO_2}} + [\mathrm{H^+}]} \cdot [\mathrm{TNN}]$ (6)	Nitric acid concentration as a function of pH, dissociation constant and total nitrite nitrogen (TNN)	[17]

Table 2: **Growth terms**

To produce more concise formulas, substrate-limited/inhibited growth, additional inhibitions, oxygen-limited growth and pH dependent growth will be represented by the formulas listed in this table. As ammonia and nitric acid were not explicitly, their concentrations is a function of their dissociation constant (temperature dependent), the pH of the medium and the total ammonia/nitrite nitrogen concentration (TAN, TNN). S is the substrate (e.g. NH_3,..), X represents the microbial species *Nitrosomonas* (Ns) and *Nitrobacter* (Nb), and "i" is the inhibiting compound (e.g. NH_3,..).

Equation	Description	Refs
$$\frac{dX_1}{dt} = (\mu_{max1} \cdot \mathcal{S}^{Ns}_{NH_3} \cdot \mathcal{I}^{Ns}_{HNO_2} \cdot \mathcal{L}^{Ns}_{O_2} \cdot \mathcal{L}_{pH} - k_{d_1})X_1 \quad (7)$$	rate of change of *Nitrosomonas* population (X_1) in co-culture	[13, 17, 17, 26, 26–30]
$$\frac{dX_2}{dt} = (\mu_{max2} \cdot \mathcal{S}^{Nb}_{HNO_2} \cdot \mathcal{I}^{Nb}_{NH_3} \cdot \mathcal{L}^{Nb}_{O_2} \cdot \mathcal{L}_{pH} - k_{d_2})X_2 \quad (8)$$	rate of change of *Nitrobacter* population (X_2) in co-culture	[13, 17, 19, 29, 31–37]
$$\frac{dTAN}{dt} = D([TAN]_{in} - [TAN]) - (\frac{\mu_{max1}}{Y_{NH_3}} + m_1) \cdot X_1 \cdot \mathcal{S}^{Ns}_{NH_3} \cdot \mathcal{I}^{Ns}_{HNO_2} \cdot \mathcal{L}^{Ns}_{O_2} \cdot \mathcal{L}_{pH} \quad (9)$$	rate of change of the total ammonium nitrogen (TAN) in co-culture	[5, 15, 15, 22, 38, 38–41]
$$\frac{dTNN}{dt} = (\frac{\mu_{max1}}{Y_{NH_3}} + m_1) \cdot X_1 \cdot \mathcal{S}^{Ns}_{NH_3} \cdot \mathcal{I}^{Ns}_{HNO_2} \cdot \mathcal{L}^{Ns}_{O_2} \cdot \mathcal{L}_{pH} - (\frac{\mu_{max2}}{Y_{HNO_2}} + m_2) \cdot X_2 \cdot \mathcal{S}^{Nb}_{HNO_2} \cdot \mathcal{I}^{Nb}_{NH_3} \cdot \mathcal{L}^{Nb}_{O_2} \cdot \mathcal{L}_{pH} - D[TNN] \quad (10)$$	rate of change of total nitrite nitrogen (TNN) in co-culture	[5, 15, 15, 22, 38, 38–41]
$$\frac{dTNNa}{dt} = (\frac{\mu_{max2}}{Y_{HNO_2}} + m_2) \cdot X_2 \cdot \mathcal{S}^{Nb}_{HNO_2} \cdot \mathcal{I}^{Nb}_{NH_3} \cdot \mathcal{L}^{Nb}_{O_2} \cdot \mathcal{L}_{pH} - D[TNNa] \quad (11)$$	rate of change of total nitrate nitrogen ($TNNa = N - NO_3^- + N - HNO_3$) in co-culture	[5, 15, 15, 22, 38, 38–41]
$$\frac{dO_2}{dt} = K_La \cdot ([O_{2sat}] - [O_2]) - \frac{3}{2}(\frac{\mu_{max1}}{Y_{NH_3}} + m_1) \cdot X_1 \cdot \mathcal{S}^{Ns}_{NH_3} \cdot \mathcal{I}^{Ns}_{HNO_2} \cdot \mathcal{L}^{Ns}_{O_2} \cdot \mathcal{L}_{pH} - \frac{1}{2}(\frac{\mu_{max2}}{Y_{HNO_2}} + m_2) \cdot X_2 \cdot \mathcal{S}^{Nb}_{HNO_2} \cdot \mathcal{I}^{Nb}_{NH_3} \cdot \mathcal{L}^{Nb}_{O_2} \cdot \mathcal{L}_{pH} - D[O_2] \quad (12)$$	rate of oxygen consumption in co-culture	[1, 42, 43]

Table 3: **Co-culture**

The system of ODEs defined in this table, describes the temporal evolution of both microbial populations (*Nitrosomonas* and *Nitrobacter*) as well as the nitrogen species (TAN, TNN and TNNa) and oxygen (O_2) involved in their metabolism, in co-culture.

Equation	Description	Refs
$$\frac{dX_1}{dt} = (\mu_{max1} \cdot \mathcal{S}^{Ns}_{NH_3} \cdot \mathcal{I}^{Ns}_{HNO_2} \cdot \mathcal{L}^{Ns}_{O_2} \cdot \mathcal{L}_{pH} - k_{d_1})X_1 \quad (13)$$	rate of change of *Nitrosomonas* population in the first bioreactor	[13, 17, 17, 26, 26–30]
$$\frac{dTAN}{dt} = D([TAN]_{in} - [TAN]) - (\frac{\mu_{max1}}{Y_{NH_3}} + m_1) \cdot X_1 \cdot \mathcal{S}^{Ns}_{NH_3} \cdot \mathcal{I}^{Ns}_{HNO_2} \cdot \mathcal{L}^{Ns}_{O_2} \cdot \mathcal{L}_{pH} \quad (14)$$	rate of change of the total ammonium nitrogen (TAN) in the first bioreactor	[5, 15, 15, 22, 38, 38–41]
$$\frac{dTNN}{dt} = (\frac{\mu_{max1}}{Y_{NH_3}} + m_1) \cdot X_1 \cdot \mathcal{S}^{Ns}_{NH_3} \cdot \mathcal{I}^{Ns}_{HNO_2} \cdot \mathcal{L}^{Ns}_{O_2} \cdot \mathcal{L}_{pH} - D[TNN] \quad (15)$$	rate of change of total nitrite nitrogen (TNN) in the first bioreactor	[5, 15, 15, 22, 38, 38–41]
$$\frac{dTNNa}{dt} = -D[TNNa] \quad (16)$$	rate of change of total nitrate nitrogen ($TNNa = N - NO_3^- + N - HNO_3$) in the first bioreactor	[5, 15, 15, 22, 38, 38–41]
$$\frac{dO_2}{dt} = K_La \cdot ([O_{2sat}] - [O_2]) - D[O_2] - X_1^{Ns} \cdot \mathcal{S}_{NH_3} \cdot \frac{3}{2}(\frac{\mu_{max1}}{Y_{NH_3}} + m_1) \cdot \mathcal{I}^{Ns}_{HNO_2} \cdot \mathcal{L}^{Ns}_{O_2} \cdot \mathcal{L}_{pH} \quad (17)$$	rate of oxygen consumption in the first bioreactor	[1, 42, 43]

Table 4: **Sequential bioreactors (first bioreactor)**

ODE system describing the first bioreactor of the sequential bioreactors scenario, where both species are cultured separately. This bioreactor only contains a monoculture of *Nitrosomonas*.

Equation	Description	Refs
$$\frac{dX_2}{dt} = (\mu_{max2} \cdot \mathcal{S}^{Nb}_{HNO_2} \cdot \mathcal{I}^{Nb}_{NH_3} \cdot \mathcal{L}^{Nb}_{O_2} \cdot \mathcal{L}_{pH} - k_{d_2})X_2 \quad (18)$$	rate of change of *Nitrobacter* population in the second bioreactor	[13, 17, 19, 29, 31–37]
$$\frac{dTAN}{dt} = D([TAN]_1 - [TAN]) \quad (19)$$	rate of change of the total ammonium nitrogen (TAN) in the second bioreactor	[5, 15, 15, 22, 38, 38–41]
$$\frac{dTNN}{dt} = D([TNN]_1 - [TNN]) - (\frac{\mu_{max2}}{Y_{HNO_2}} + m_2) \cdot X_2 \cdot \mathcal{S}^{Nb}_{HNO_2} \cdot \mathcal{I}^{Nb}_{NH_3} \cdot \mathcal{L}^{Nb}_{O_2} \cdot \mathcal{L}_{pH} \quad (20)$$	rate of change of total nitrite nitrogen (TNN) in the second bioreactor	[5, 15, 15, 22, 38, 38–41]
$$\frac{dTNNa}{dt} = D([TNNa]_1 - [TNNa]) + (\frac{\mu_{max2}}{Y_{HNO_2}} + m_2) \cdot X_2 \cdot \mathcal{S}^{Nb}_{HNO_2} \cdot \mathcal{I}^{Nb}_{NH_3} \cdot \mathcal{L}^{Nb}_{O_2} \cdot \mathcal{L}_{pH} \quad (21)$$	rate of change of total nitrate nitrogen ($TNNa = N - NO_3^- + N - HNO_3$) in the second bioreactor	[5, 15, 15, 22, 38, 38–41]
$$\frac{dO_2}{dt} = K_L a \cdot ([O_{2sat}] - [O_2]) + D([O_2]_1 - [O_2]) - \frac{1}{2}(\frac{\mu_{max2}}{Y_{HNO_2}} + m_2) \cdot X_2 \cdot \mathcal{S}^{Nb}_{HNO_2} \cdot \mathcal{I}^{Nb}_{NH_3} \cdot \mathcal{L}^{Nb}_{O_2} \cdot \mathcal{L}_{pH} \quad (22)$$	rate of oxygen consumption in the second bioreactor	[1, 42, 43]

Table 5: **Sequential bioreactors (second bioreactor)**

The second sequential bioreactor contains a monoculture of *Nitrobacter* whose population is described by the ODE listed in this table, together with the concentrations of the nitrogen species and oxygen.

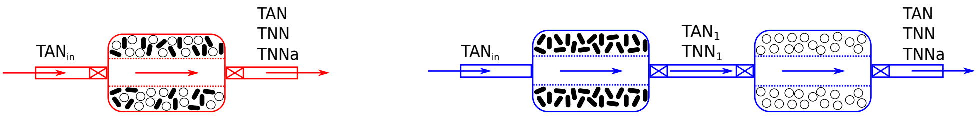

	Nitrosomonas [mg biomass/L]	*Nitrobacter* [mg biomass/L]	TAN [mg/L]	TNN [mg/L]	TNNa [mg/L]	O_2 [mg/L]	$Y_{NO3-/NH3}$ [mg N-NO_3^- produced/ mg N-NH_3 consumed]
**Coculture** pH = 8, T = 35°C	950.74	30.32	17.68 (2%)	889.61 (89%)	92.72 (9%)	0.0009 (0.01%)	9%
**Sequential** pH = 8, T = 35°C	967.38	323.96	0.16 (0%)	0.015 (0%)	999.83 (100%)	0.11/5.34 (1%/67%)	100%

Figure 1: **Co-culture vs. Sequential bioreactors: steady-state values**

This illustration shows the two layouts of bioreactors that are compared for their ability to convert ammonia to nitrate. In the co-culture (red) both microbial species are cultured together, whereas in the sequential scenario (blue) they grow in separate bioreactors. The first chamber contains *Nitrosomonas sp.* which convert ammonia (NH_3) to nitrite (NO_2^-) and its content is fed to the second bioreactor culturing *Nitrobacter sp.* which oxidizes nitrite to nitrate (NO_3^-). The steady-state values of the microbial populations, nitrogen species (total ammonium nitrogen, $TAN = N-NH_3 + N-NH_4^+$, total nitrite nitrogen, $TNN = N-HNO_2 + N-NO_2^-$ and total nitrate nitrogen, $TNNa = N-HNO_3 + N-NO_3^-$) and oxygen concentration (first and second bioreactor values) are listed for the co-culture and the sequential bioreactors (same pH and temperature as in co-culture). Microbial populations are given in [mg biomass/L] and the chemical species in [mg/L]. The percentages indicated next to the values are relative to the total nitrogen injected into the system (TAN_{in}) therefore the sum of all the nitrogen species is equal to the input concentration, here $[TAN_{in}]$ = 1000 [mg TAN/L]. The values for the nitrogen species indicated for the sequential scenario are the output concentrations of the second bioreactor (containing *Nitrobacter*).

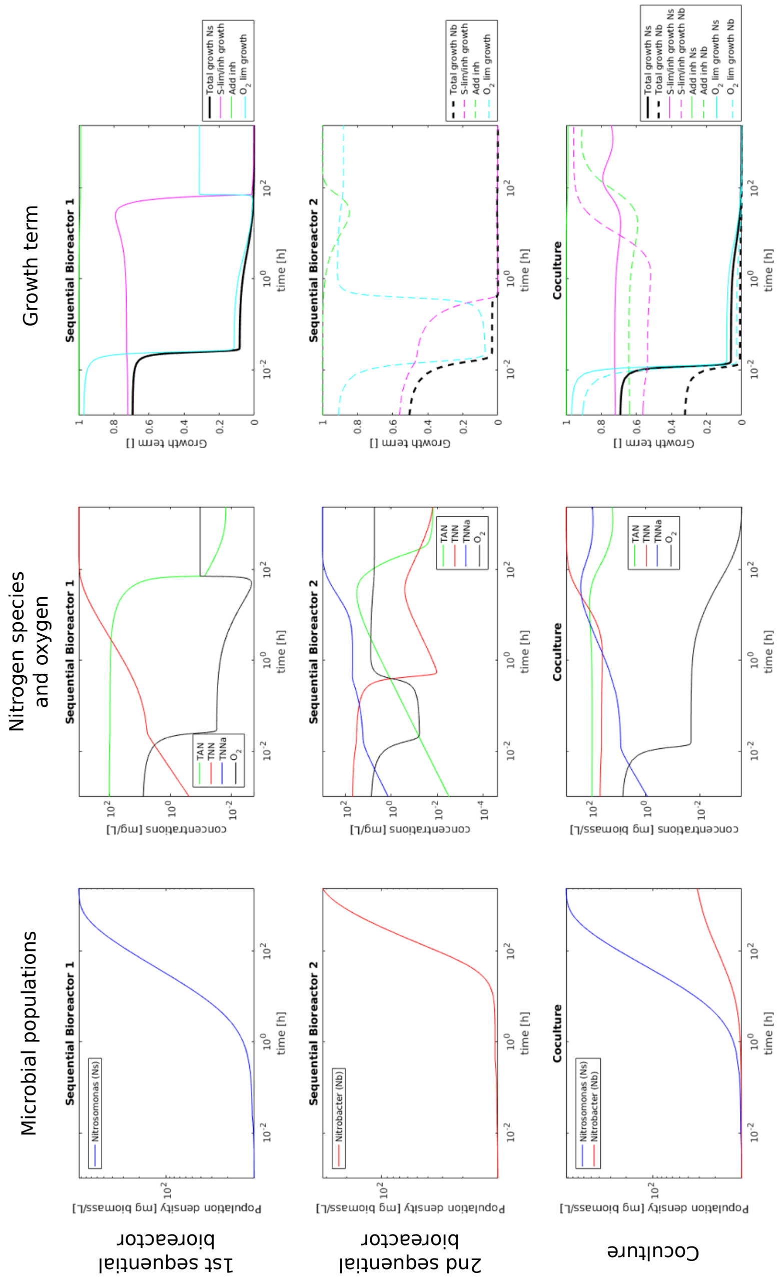

Figure 2: **Co-culture vs. Sequential bioreactors**

These plots show the dynamic behavior of both microbial populations, nitrogen species and oxygen concentrations, as well as the growth term for each microorganism for the sequential bioreactors and the co-culture. The growth term plots display the contributions of substrate-limited/inhibited growth (S-lim/inh, magenta line), additional inhibitions (Add inh, green line) and oxygen limitation (O_2 lim, cyan line) to the total growth term (thick black line). Since the pH dependence of growth is constant (due to a pH value maintained constant) and close to 1 it is not shown on the plots.

Parameter	Value	Description	Refs
μ_{max1}	$0.47 \cdot e^{0.098 \cdot (T-15)}$ (23)	Specific growth rate of *Nitrosomonas* $[h^{-1}]$	[24, 44]
μ_{max2}	$0.79 \cdot e^{0.069 \cdot (T-15)}$ (24)	Specific growth rate of *Nitrobacter* $[h^{-1}]$	[24, 44]
K_{NH_3}	$\frac{17}{14} \cdot \frac{Ka_{NH_4^+}}{Ka_{NH_4^+} + [H^+]} \cdot 10^{0.051T-1.158)}$ (25)	Affinity constant for NH_3 by *Nitrosomonas* $[mg\ NH_3/L]$	[24, 30, 44]
K_{HNO_2}	$\frac{47}{14} \cdot \frac{[H^+]}{Ka_{HNO_2} + [H^+]} \cdot 10^{0.063T-1.149)}$ (26)	Affinity constant for HNO_2 by *Nitrobacter* $[mg\ HNO_2/L]$	[24, 30, 44]
$Ka_{NH_4^+}$	$\frac{[NH_3][H^+]}{[NH_4^+]} = e^{-\frac{6344}{273+T}}$ (27)	Acid dissociation constant for NH_4^+ [mol/L]	[13]
Ka_{HNO_2}	$\frac{[NO_2^-][H^+]}{[HNO_2]} = e^{-\frac{2300}{273+T}}$ (28)	Acid dissociation constant for HNO_2 [mol/L]	[13]
Y_{NH_3}	0.097	Yield for microbial growth of *Nitrosomonas* on NH_3 [mg biomass/mg NH_3]	[20]
Y_{HNO_2}	0.012	Yield for microbial growth of *Nitrobacter* on HNO_2 [mg biomass/mg HNO_2]	[20]
m_1	0.061	Maintenance rate of *Nitrosomonas* [mg NH_3/(mg biomass · h)]	[20]
m_2	0.365	Maintenance rate of *Nitrobacter* [mg HNO_2/(mg biomass · h)]	[20]
$K_{I_{NH_3}}^{Ns}$	30.24	Substrate inhibition of *Nitrosomonas* [mg NH_3/L]	[17]
$K_{I_{HNO_2}}$	1.48	Inhibition of *Nitrosomonas* by HNO_2 [mg HNO_2/L]	[17]
$K_{I_{HNO_2}}^{Nb}$	7.755	Substrate inhibition of *Nitrobacter* [mg HNO_2/L]	[17]
$K_{I_{NH_3}}$	17.97	Inhibition of *Nitrobacter* by NH_3 [mg NH_3/L]	[17]
k_{d1}	0.003	Death rate of *Nitrosomonas* $[h^{-1}]$	[20]
k_{d2}	0.001	Death rate of *Nitrobacter* $[h^{-1}]$	[20]
D	0.03	Dilution rate $[h^{-1}]$	[20]
$[TAN]_{in}$	1000	Concentration of TAN ($[N-NH_3] + [N-NH_4^+]$) in the feed [mg TAN/L]	[20]
$[O_{2sat}]$	8	Oxygen saturation concentration [mg O_2/L]	[42]
K_La	5.7	Volumetric oxygen transfer coefficient $[h^{-1}]$ (set manually in order to reach oxygen limitation in the first sequential bioreactor)	
$K_{O_2}^{Ns}$	0.23	Affinity constant for oxygen by *Nitrosomonas* [mg O_2/L]	[17]
$K_{O_2}^{Nb}$	0.73	Affinity constant for oxygen by *Nitrobacter* [mg O_2/L]	[17]
K_{pH}	26.6	pH constant [scalar]	[17]
pH_{co}	8	pH in the co-culture [mol/L] (chosen as intermediate value between pH optima from Grunditz et al.	[45])
pH_{opt1}	8.1	Optimal growth pH for *Nitrosomonas* [mol/L]	[45]
pH_{opt2}	7.9	Optimal growth pH for *Nitrobacter* [mol/L]	[45]
T_{co}	35	Temperature in the co-culture [°C]	[45]
T_1	35	Optimal growth temperature for *Nitrosomonas* [°C]	[45]
T_2	38	Optimal growth temperature for *Nitrobacter* [°C]	[45]

Table 6: **Parameters values, units and definitions**

The parameter values listed here are extracted from the literature cited on the right. Despite the fact that some "parameters" are temperature dependent, we chose to list them here as each experiment was simulated at constant temperature, and thus the parameter was a constant.

Initial values	Value	Description
$X1_0$	10	Initial population of *Nitrosomonas* in both scenarios [mg biomass/L]
$X2_0$	10	Initial population of *Nitrobacter* in both scenarios [mg biomass/L]
$[TAN]_0$	100	Initial concentration of TAN (total ammonium nitrogen = $[N-NH_3]+[N-NH_4^+]$) in the co-culture [mg TAN/L]
$[TNN]_0$	50	Initial concentration of TNN (total nitrite nitrogen = $[N-HNO_2]+[N-NO_2^-]$) in the co-culture [mg TNN/L]
$[TNNa]_0$	0	Initial concentration of TNNa (total nitrate nitrogen = $[N-HNO_3]+[N-NO_3^-]$) in the co-culture [mg TNNa/L]
$[O_2]_0$	8	Initial concentration of O_2 in the co-culture [mg O_2/L]
$[TAN]1_0$	100	Initial concentration of TAN (total ammonium nitrogen = $[N-NH_3]+[N-NH_4^+]$) in the 1st sequential bioreactor [mg TAN/L]
$[TNN]1_0$	0	Initial concentration of TNN (total nitrite nitrogen = $[N-HNO_2]+[N-NO_2^-]$) in the 1st sequential bioreactor [mg TNN/L]
$[TNNa]1_0$	0	Initial concentration of TNNa (total nitrate nitrogen = $[N-HNO_3]+[N-NO_3^-]$) in the 1st sequential bioreactor [mg TNNa/L]
$[O_2]1_0$	8	Initial concentration of O_2 in the 1st sequential bioreactor [mg O_2/L]
$[TAN]2_0$	0	Initial concentration of TAN (total ammonium nitrogen = $[N-NH_3]+[N-NH_4^+]$) in the 2nd sequential bioreactor [mg TAN/L]
$[TNN]2_0$	50	Initial concentration of TNN (total nitrite nitrogen = $[N-HNO_2]+[N-NO_2^-]$) in the 2nd sequential bioreactor [mg TNN/L]
$[TNNa]2_0$	0	Initial concentration of TNNa (total nitrate nitrogen = $[N-HNO_3]+[N-NO_3^-]$) in the 2nd sequential bioreactor [mg TNNa/L]
$[O_2]2_0$	8	Initial concentration of O_2 in the 2nd sequential bioreactor [mg O_2/L]

Table 7: **Initial conditions**

The initial values did not have much of an impact on the steady-state values of the system and they were chosen so as to allow for a smooth transition towards the steady-state values (i.e. limit initial population decline by lack of substrate or inhibiting concentrations of substrate or other inhibitors). The same substrates concentrations were applied to the co-culture and sequential bioreactors. The initial oxygen concentration was set to its saturation value $[O_{2sat}]$. The first sequential bioreactor contained TAN in the original medium but no TNN, and vice versa for the second bioreactor.

References

[1] D. Dochain and P. A. Vanrolleghem, *Dynamical Modelling and Estimation in Wastewater Treatment Processes.* London: IWA Publishing, 2001.

[2] M. Henze, P. Harremoës, J. Cour Jansen, and E. Arvin, *Wastewater Treatment.* Berlin, Heidelberg: Springer Berlin Heidelberg, 1997.

[3] C. P. L. et al. Grady, *Biological Wastewater Treatment.* London: IWA Publishing, 3rd ed., 2011.

[4] A. van Haandel and J. van der Lubbe, *Handbook of Biological Wastewater Treatment: Design and Optimisation of Activated Sludge Systems.* IWA Publishing, 2012.

[5] Metcalf and Eddy, *Wastewater engineering: treatment and reuse.* Boston: McGraw-Hill, 4th ed., 2003.

[6] D. J. Arp, L. A. Sayavedra-Soto, and N. G. Hommes, “Molecular biology and biochemistry of ammonia oxidation by Nitrosomonas europaea,” *Archives of Microbiology*, vol. 178, no. 4, pp. 250–255, 2002.

[7] C. Buchwald, A. E. Santoro, M. R. McIlvin, and K. L. Casciotti, "Oxygen isotopic composition of nitrate and nitrite produced by nitrifying cocultures and natural marine assemblages," *Limnology and Oceanography*, vol. 57, no. 3, pp. 1361–1375, 2012.

[8] U. E. P. Agency, "Nitrification," tech. rep., U.S. Environmental Protection Agency, 2002.

[9] J. I. Prosser, "Nitrogen in Soils: Nitrification," *The Encyclopedia of Soils in the Environment*, p. 292, 2005.

[10] B. Ward, "Nitrification," in *Reference Module in Earth Systems and Environmental Sciences*, pp. 1–8, Elsevier, 2013.

[11] U. Wiesmann, "Biological nitrogen removal from wastewater," in *Advances in Biochemical Engineering/Biotechnology*, vol. 51, pp. 113–154, Weinheim, Germany: Wiley-VCH Verlag GmbH & Co. KGaA, oct 1994.

[12] H. Painter, "A review of literature on inorganic nitrogen metabolism in microorganisms," *Water Research*, vol. 4, pp. 393–450, jun 1970.

[13] A. C. Anthonisen, R. C. Loehr, T. B. Prakasam, and E. G. Srinath, "Inhibition of nitrification by ammonia and nitrous acid.," *Journal - Water Pollution Control Federation*, vol. 48, pp. 835–52, may 1976.

[14] J. Haldane, *Enzymes.* New York: Longmans, Green, 1930.

[15] S. Pirt, *Principles of microbe and cell cultivation.* Oxford: Blackwell, 1975.

[16] A. Mulchandani and J. Luong, "Microbial inhibition kinetics revisited," *Enzyme and Microbial Technology*, vol. 11, pp. 66–73, feb 1989.

[17] A. Magrí, L. Corominas, H. López, E. Campos, M. Balaguer, J. Colprim, and X. Flotats, "A Model for the Simulation of the SHARON Process: pH as a Key Factor," *Environmental Technology*, vol. 28, no. 3, pp. 255–265, 2007.

[18] J. F. Andrews, "A mathematical model for the continuous culture of microorganisms utilizing inhibitory substrates," *Biotechnology and Bioengineering*, vol. 10, pp. 707–723, nov 1968.

[19] B. Boon and H. Laudelout, "Kinetics of nitrite oxidation by Nitrobacter winogradskyi," *Biochemical Journal*, vol. 85, pp. 440–447, dec 1962.

[20] A. Montras Boet, *Mathematical modelling and molecular analysis of a nitrifying packed bed biofilm reactor.* PhD thesis, Universitat Autonoma de Barcelona, 2009.

[21] S. Aiba, M. Shoda, and M. Nagatani, "Kinetics of product inhibition in alcohol fermentation," *Biotechnology and Bioengineering*, vol. 10, pp. 845–864, nov 1968.

[22] J. Monod, *Recherches sur la croissance des cultures bactériennes.* Paris: Hermann, 1942.

[23] J. Monod, "The Growth of Bacterial Cultures," *Annual Review of Microbiology*, vol. 3, pp. 371–394, oct 1949.

[24] U.S. Environmental Protection Agency, "Process Design Manual for Nitrogen Control," tech. rep., U.S. Environmental Protection Agency, 1975.

[25] U.S. Environmental Protection Agency, "Manual: Nitrogen Control," tech. rep., U.S. Environmental Protection Agency, 1993.

[26] S. W. Van Hulle, E. I. Volcke, J. L. Teruel, B. Donckels, M. C. van Loosdrecht, and P. A. Vanrolleghem, "Influence of temperature and pH on the kinetics of the Sharon nitritation process," *Journal of Chemical Technology & Biotechnology*, vol. 82, pp. 471–480, may 2007.

[27] V. M. Vadivelu, J. Keller, and Z. Yuan, "Stoichiometric and kinetic characterisation of Nitrosomonas sp. in mixed culture by decoupling the growth and energy generation processes," *Journal of Biotechnology*, vol. 126, pp. 342–356, nov 2006.

[28] V. M. Vadivelu, J. Keller, and Z. Yuan, "Effect of free ammonia and free nitrous acid concentration on the anabolic and catabolic processes of an enriched Nitrosomonas culture," *Biotechnology and Bioengineering*, vol. 95, pp. 830–839, dec 2006.

[29] C. Hellinga, M. van Loosdrecht, and J. Heijnen, "Model Based Design of a Novel Process for Nitrogen Removal from Concentrated Flows," *Mathematical and Computer Modelling of Dynamical Systems*, vol. 5, pp. 351–371, dec 1999.

[30] S. Park and W. Bae, "Modeling kinetics of ammonium oxidation and nitrite oxidation under simultaneous inhibition by free ammonia and free nitrous acid," *Process Biochemistry*, vol. 44, pp. 631–640, jun 2009.

[31] J. Chung, H. Shim, S.-J. Park, S.-J. Kim, and W. Bae, "Optimization of free ammonia concentration for nitrite accumulation in shortcut biological nitrogen removal process," *Bioprocess and Biosystems Engineering*, vol. 28, pp. 275–282, mar 2006.

[32] W. Bae, S. Baek, J. Chung, and Y. Lee, "Optimal operational factors for nitrite accumulation in batch reactors," *Biodegradation*, vol. 12, no. 5, pp. 359–366, 2001.

[33] C. S. Gee, J. T. Pfeffer, and M. T. Suidan, "Nitrosomonas and Nitrobacter Interactions in Biological Nitrification," *Journal of Environmental Engineering*, vol. 116, pp. 4–17, feb 1990.

[34] C. S. Gee, M. T. Suidan, and J. T. Pfeffer, "Modeling of Nitrification Under SubstrateInhibiting Conditions," *Journal of Environmental Engineering*, vol. 116, pp. 18–31, feb 1990.

[35] K.-I. Gil and E.-S. Choi, "Modelling of inhibition of nitrite oxidation in biological nitritation processes by free ammonia," *Biotechnology Letters*, vol. 23, no. 24, pp. 2021–2026, 2001.

[36] V. M. Vadivelu, Z. Yuan, C. Fux, and J. Keller, "The Inhibitory Effects of Free Nitrous Acid on the Energy Generation and Growth Processes of an Enriched Nitrobacter Culture," *Environmental Science & Technology*, vol. 40, pp. 4442–4448, jul 2006.

[37] V. M. Vadivelu, J. Keller, and Z. Yuan, "Effect of free ammonia on the respiration and growth processes of an enriched Nitrobacter culture," *Water Research*, vol. 41, pp. 826–834, feb 2007.

[38] S. J. Pirt, "The Maintenance Energy of Bacteria in Growing Cultures," *Proceedings of the Royal Society of London. Series B, Biological Sciences*, vol. 163, no. 991, pp. 224–231, 1965.

[39] J. E. Bailey and D. F. Ollis, *Biochemical Engineering Fundamentals*. New York: McGraw-Hill, 2nd ed., 1986.

[40] K. Kovárová-Kovar and T. Egli, "Growth kinetics of suspended microbial cells: from single-substrate-controlled growth to mixed-substrate kinetics.," *Microbiology and molecular biology reviews : MMBR*, vol. 62, pp. 646–66, sep 1998.

[41] K. L. Schulze and R. S. Lipe, "Relationship between substrate concentration, growth rate, and respiration rate of Escherichia coli in continuous culture," *Archiv fuer Mikrobiologie*, vol. 48, no. 1, pp. 1–20, 1964.

[42] R. Battino, H. Clever, and C. Young, "THE SOLUBILITY OF GASES IN LIQUIDS," in *Oxygen and Ozone*, vol. 7, pp. xiii–xviii, Elsevier, 1981.

[43] P. Hagander and M. Akesson, "Control of Dissolved Oxygen in Stirred Bioreactors," tech. rep., Department of Automatic Control, Lund University, Sweden, 2000.

[44] G. Knowles, A. L. Downing, and M. J. Barrett, "Determination of Kinetic Constants for Nitrifying Bacteria in Mixed Culture, with the Aid of an Electronic Computer," *Journal of General Microbiology*, vol. 38, pp. 263–278, feb 1965.

[45] C. Grunditz and G. Dalhammar, "Development of nitrification inhibition assays using pure cultures of nitrosomonas and nitrobacter," *Water Research*, vol. 35, pp. 433–440, feb 2001.
